# Precision Nanoconfined Self‐Assembly of ACQ Carbon Dots for Enhanced Solid‐State Fluorescence

**DOI:** 10.1002/advs.202503317

**Published:** 2025-05-08

**Authors:** Jingyi Hao, Wenjie Zhang, Yuying Li, Wenjun Ma, Yueying Zhu, Junle Zhang, Ge Shi, Xiaoguang Qiao, Yanjie He, Zheng Zhao, Xinchang Pang, Ben Zhong Tang

**Affiliations:** ^1^ Henan Joint International Research Laboratory of Living Polymerizations and Functional Nanomaterials Henan Key Laboratory of Advanced Nylon Materials and Application School of Materials Science and Engineering Zhengzhou University Zhengzhou 450001 P. R. China; ^2^ School of Materials Science and Engineering School of Chemistry & Chemical Engineering Henan University of Science and Technology Luoyang 471023 P. R. China; ^3^ School of Science and Engineering Shenzhen Institute of Aggregate Science and Technology Shenzhen Key Laboratory of Functional Aggregate Materials The Chinese University of Hong Kong Shenzhen Guangdong 518172 P. R. China; ^4^ Department of Chemistry and the Hong Kong Branch of Chinese National Engineering Research Center for Tissue Restoration and Reconstruction The Hong Kong University of Science and Technology Clear Water Bay Kowloon Hong Kong 999077 P. R. China

**Keywords:** aggregation induced emission, block copolymers, carbon dots, micelles, solid‐state fluorescence

## Abstract

Carbon dots (CDs) are promising fluorescent nanomaterials, however, they are often hindered by aggregation caused quenching (ACQ) in solid‐state application because of close π–π stacking interactions. Furthermore, the challenges still exist in the development of CDs‐based solid‐state fluorescent materials with stable structure and high fluorescence intensity. To address this challenge, a general and robust polymer directed nanoconfined self‐assembly strategy is developed, enabling the fabrication of regular morphology, structurally ultra‐stable and solid‐state fluorescent CDs assemblies using hydrophilic star‐liked di‐block copolymer unimolecular micelles as templates. The absolute photoluminescence quantum yield (PLQY) of these fluorescent solid‐state CD assemblies reaches 21.46%, significantly higher than 0.12% observed in traditional ACQ solid‐state CDs. The enhanced solid‐state fluorescent property is attributed to the prevention of the π–π stacking of CDs, the restricted movement of surface groups and the suppression of non‐radiative transition processes via the polymer directed nanoconfined self‐assembly of CDs. The fluorescence intensity of CDs assemblies can also be precisely tuned by adjusting the polymerization time of polymer template. Based on these advantages, the CDs assemblies are employed as luminescent materials in the identification of latent fingerprints (LFP), flexible films and 3D printing functional hydrogels.

## Graphical abstract

Star‐liked polymer unimolecular micelles are used as a general and robust nanoconfined scaffold template to fabricate structurally ultra‐stable and solid‐state fluorescence CDs assemblies via the conduction of ACQ‐to‐Solid state fluorescence transition process with ACQ CDs as the building units, which realized potential applications in detecting latent fingerprints, flexible films, and 3D printing.

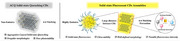

## 1 Introduction

Carbon dots (CDs) have proven to be a great promising class of fluorescent materials due to their particular electronic and optical characteristic, including tunable emission spectra, broad excitation band, biocompatibility, and nontoxicity.^[^ ^1^, ^2^, ^3^ ^]^ These characteristics endow CDs with various applications in a wide variety fields, such as bioimaging,^[^ ^4^ ^]^ sensors,^[^ ^5^ ^]^ optoelectronics,^[^ ^6^ ^]^ and photocatalysis.^[^ ^7^ ^]^ However, the majority of CDs are suffered with aggregation‐caused quenching (ACQ) because of close π–π stacking that severely limit their application in high concentration or solid‐state form.^[^ ^8^, ^9^, ^10^ ^]^ Currently, the common approach to solve the ACQ issues involves doping and dispersing CDs in matrices,^[^ ^11^, ^12^, ^13^ ^]^ such as starch, microcrystalline cellulose, inorganic salt, and silica gel to form composite materials, or grafting high steric hindrance polymer chain groups on the surface of CDs,^[^ ^14^, ^15^, ^16^ ^]^ thus, π–π stacking and transfer of non‐radiation energy could be inhibited. Still, these methods often result in uneven dispersion of CDs in the matrix, unable to be used without matrix, and the process is cumbersome and costly.

Since the aggregation induced emission (AIE) proposed by Tang et al. in 2001,^[^ ^17^ ^]^ AIE has been widely used to produce fluorescent materials for constructing optoelectronic, biosensing and bioimaging devices. The occurrence mechanism of AIE effect is attributed to the restriction of intramolecular motion (RIM) within the aggregates of AIE molecules.^[^ ^18^, ^19^ ^]^ Thus, these novel luminescent materials are weakly or non‐fluorescent in dispersed state, but exhibit bright luminescence when aggregation, which contrasting directly with the ACQ phenomenon. In the past decades, numerous luminescent materials with AIE properties have been developed, including metal complex, organic molecules, polymers, and various metal nanoclusters, etc. Inspired by AIE mechanism, it is meaningful to prepare solid‐state fluorescent CDs nanomaterials via the nanoconfinement effect to prevent the π–π stacking and vibration of CDs. The direct synthesis of CDs with AIE characteristics has been an ideal solution to address the ACQ problem. However, while numerous studies have explored the synthesis of CDs with AIE characteristics, there remain several challenges persist. i) The structural requirements for carbon precursors are stringent, thereby restricting the variety of CDs.^[^ ^20^, ^21^ ^]^ ii) CDs with AIE characteristics often have low absolute PLQY, which limits their practical application as luminescent materials.^[^ ^22^, ^23^ ^]^ iii) Most CDs are inherently hydrophobic and possess irregular morphologies, resulting in weak intermolecular interactions among them, consequently leading to their instability in complex environments.^[^ ^24^, ^25^ ^]^ Hence, it is significant to prepare hydrophilic CDs assemblies with highly efficient solid‐state fluorescence, regular morphology, and excellent stability through a general and robust strategy.

Nanoreactor strategies, in which precursors or nanoparticles are nanoconfined in a limited nanosized volumes, then in situ generated or assembled into nanomaterials, have been emerged as a significant tool for the preparation of nanoparticles with adjustable size, composition, and morphology.^[^ ^26^, ^27^ ^]^ Compared with other polymer‐based nanoreactors (such as linear block polymers micelles, dendrimers), unimolecular micelles exhibit superior stability, adjustable size and flexible reaction sites.^[^ ^28^ ^]^ Our recent studies have explored the synthesis of various functional nanomaterials using unimolecular micelles, which provide a stable growth environment and impart the nanomaterials with novel functionalities and properties from the polymers.^[^ ^29^, ^30^, ^31^ ^]^

Herein, a versatile and simple strategy was proposed to utilize hydrophilic star‐liked polymer β‐cyclodextrin‐*grafted*‐poly(acrylic acid)‐*block*‐poly(oligo(ethylene glycol) acrylate) (β‐CD‐*g*‐PAA‐*b*‐POEGA) unimolecular micelles as nanoreactors for the polymer directed nanoconfined self‐assembly of ACQ CDs to prepare solid‐state fluorescent CDs assemblies with well‐defined morphology, ultra‐stable and tunable fluorescence intensity properties. In this system, the CDs assemblies can indeed be regarded as nanoconfined aggregates, where the CDs are spatially separated by PAA chains. Unlike conventional doping strategies that disperse CDs at low density within a passive matrix, this approach creates a high‐density yet isolated assembly through surface modification with PAA‐*b*‐POEGA polymers. The PAA chains act as both electrostatic binders and physical spacers, effectively preventing direct π–π stacking between adjacent CDs and also enhanced the emission via the restricted intramolecular motion of CDs. The CDs assemblies possess an excellent application prospect in fingerprint recognition, flexible films and 3D printing in the solid‐state. Moreover, the AIE mechanism and template‐regulated fluorescence enhancement provide new insights for the design of solid‐state luminescent materials.

## 2 Results and Discussion

**Scheme 1:**
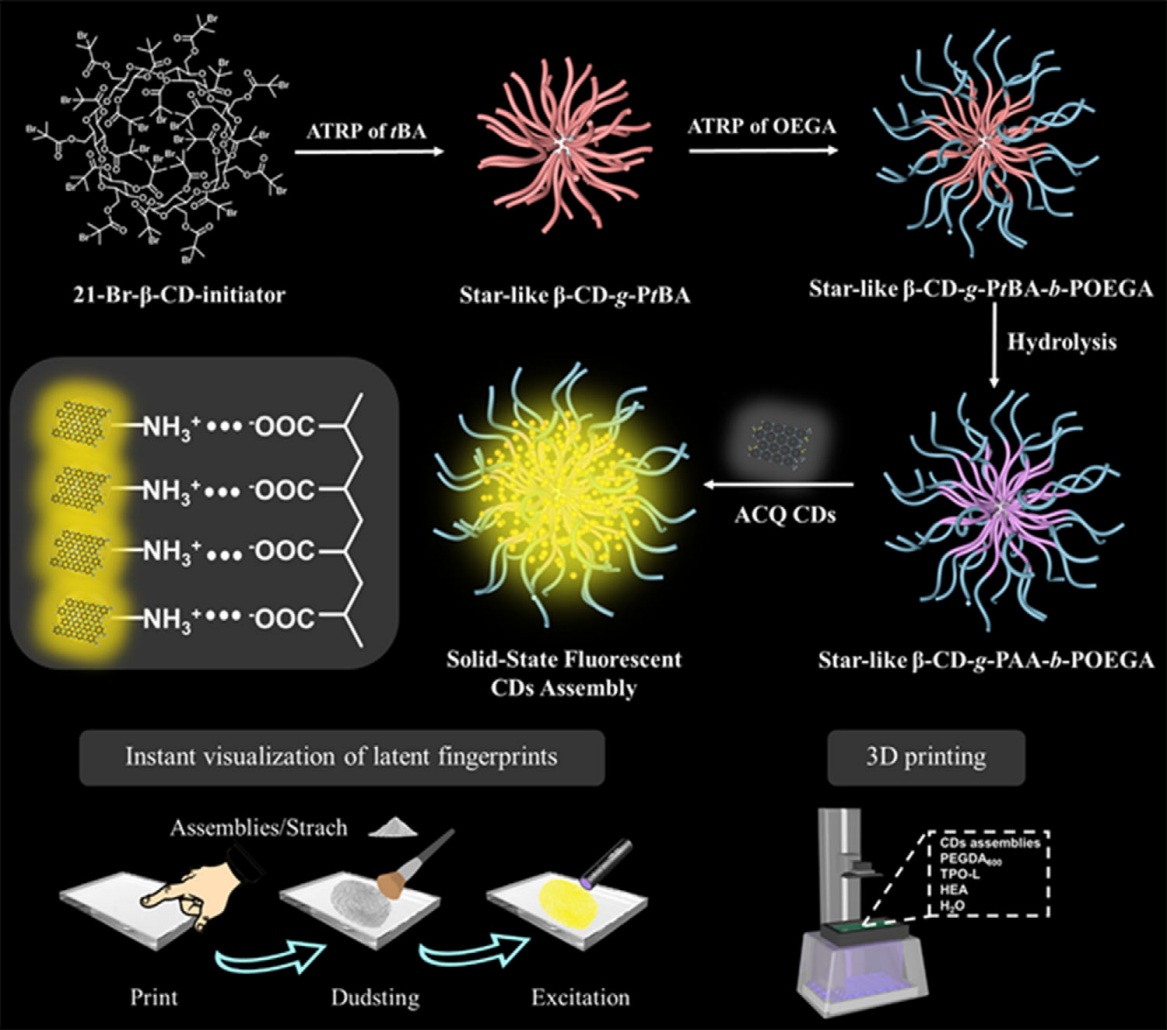
Schematic of the polymer unimolecular micelles directed nanoconfined self‐assembly of ACQ CDs with star‐liked hydrophilic polymer β‐CD‐*g*‐PAA‐*b*‐POEGA as templates scaffolds and its application in instant visualization of LFP and 3D printing.

**Scheme** **1** illustrates the synthesis strategy of fluorescent CDs assemblies with multi‐arm star‐liked polymer hydrophilic β‐CD‐*g*‐PAA‐*b*‐POEGA unimolecular micelles as scaffold templates. First, the 21 hydroxyl groups of β‐CD (β‐cyclodextrin) were esterified into bromine functional groups for the synthesis of 21‐Br‐β‐CD macroinitiators with 21 multiple atom transfer radical polymerization (ATRP)initiating sites (Figures S1 and S2, Supporting Information). Subsequently, β‐cyclodextrin‐*grafted*‐poly(*tert*‐butyl acrylate)‐*block*‐poly(oligo(ethylene glycol) acrylate) (β‐CD‐*g*‐P*t*BA‐*b*‐POE star‐liked di‐block copolymers with controlled molecular weights and narrow dispersity was synthesized via an efficient photoinduced ATRP process.^[^ ^32^, ^33^ ^]^ The GPC curves of star‐liked polymers exhibited symmetric monomodal peaks with narrow molecular weight distribution, indicating the high controllability of polymerization (Tables S1 and S2 and Figures S3–S6, Supporting Information). Then, the P*t*BA domain was completely hydrolyzed to polyacrylic acid (PAA) by trifluoroacetic acid (TFA), yielding the final hydrophilic β‐CD‐*g*‐PAA‐*b*‐POEGA polymer unimolecular micelles (Figures S4 and S7, Supporting Information), which was possessed as the nanoconfined nanoreactors for the directed self‐assembly of CDs.

**Figure 1:**
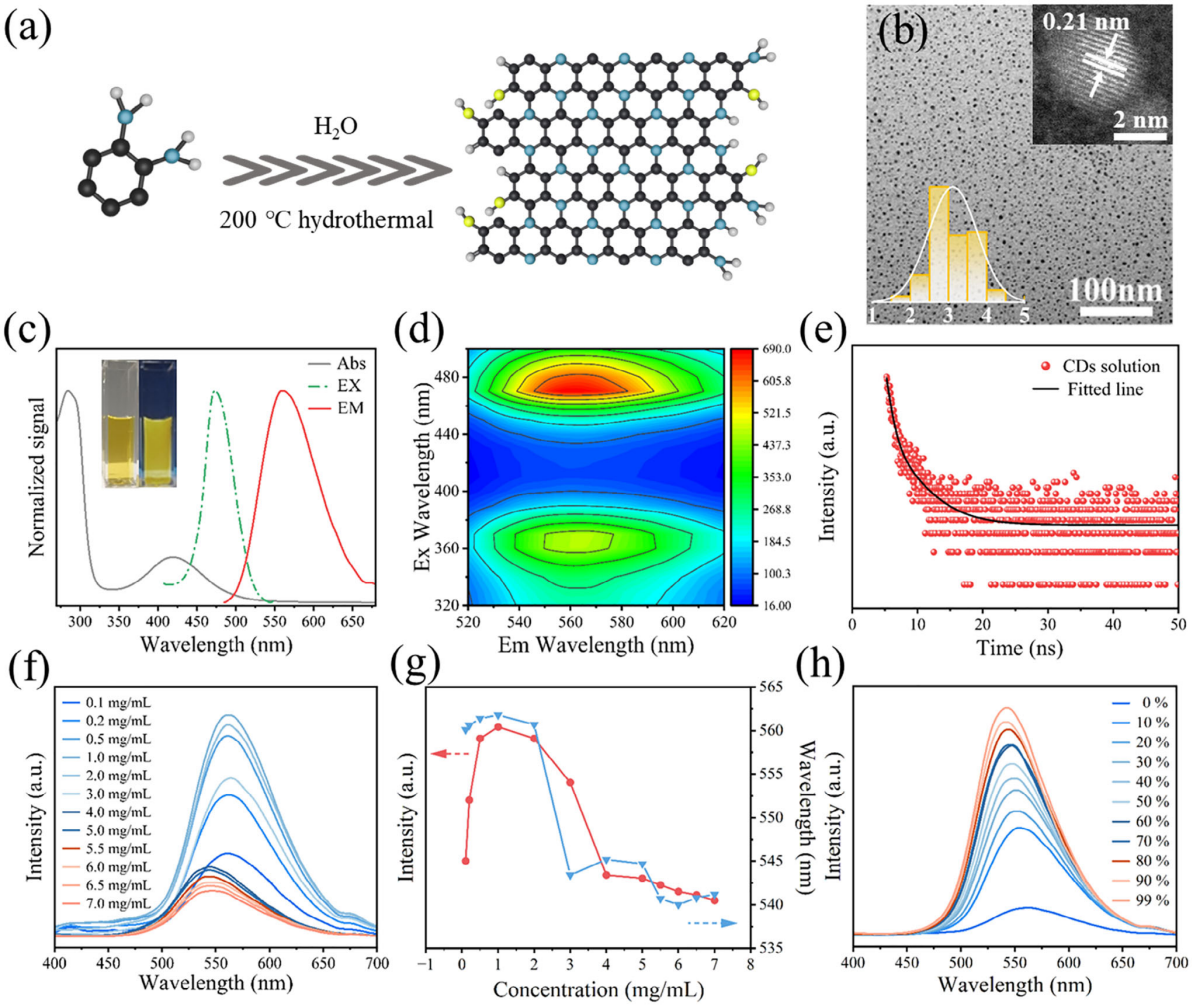
a) Synthesis procedure of CDs; b) TEM image of CDs, insets show size distribution and HRTEM image of the CDs; c) UV–vis absorption (gray line), emission spectra (solid red lines, *λ*_ex_ = 470 nm), and photoexcitation (green dot lines, *λ*_em_ = 560 nm) spectrum of CDs solution, insets show digital photo of CDs in aqueous solution under sunlight and UV light; d) The contour map of fluorescence spectrum of CDs solution; e) Fluorescence decay curve of CDs solution; f) Fluorescence spectrum of the CDs in aqueous solution (concentrations ranging from 0.1 to 7 mg mL^−1^); g) Curve of fluorescence intensity and wavelength of CDs aqueous solution with different concentration; h) Fluorescence spectrum of the CDs solution with different ratios of glycerol in glycerol/water mixed solvent.

With o‐phenylenediamine as precursor, yellow emissive CDs were synthesized via hydrothermal method (**Figure** **1**a). The transmission electron microscope (TEM) images showed that these CDs were monodispersed nanoparticles with diameter ranging about ≈3.0 nm, High‐resolution TEM (HRTEM) of CDs shows lattice spacing is about 0.21 nm, attributed to the (100) plane of graphite (Figure 1b).^[^ ^34^ ^]^ In addition, the X‐ray diffraction (XRD) pattern of CDs depicts an obvious broad diffraction peak at 23°, which correspond to the (002) of the graphite structure (Figure S8, Supporting Information).^[^ ^35^ ^]^ Two vibration peaks at 1368 and 1530 cm^−1^ were observed in the Raman spectrum of CDs under the excitation of 532 nm light, representing the disordered (D‐band) and graphite (G‐band) of CDs’ frameworks, respectively (Figure S9, Supporting Information). The intensity ratio *I*_D_/*I*_G_ is 0.92 indicating that the prepared CDs had graphite structural properties.^[^ ^36^ ^]^ The normalized Fourier transform‐infrared (FT‐IR) spectrum of CDs (Figure S10, Supporting Information) confirmed the presence of O─H (3384 cm^−1^), N─H (3190 cm^−1^), NH_2_ (1500 cm^−1^), and C═O (1635 cm^−1^). The full X‐ray photoelectron spectroscopy (XPS) spectrum of CDs have peaks at 281, 396, and 529 eV corresponding to C 1s, N 1s, and O 1s, respectively. In the high‐resolution XPS (Figure S11, Supporting Information), the C 1s spectra can be deconvoluted in three distinct peaks: 284.1 eV (C−C/C═C), 285.1 eV (C−N/C─OH), 288.1 eV (C−O/C═O), respectively. The N 1s spectra displays peak at 398.6 eV, can be attributed to the pyridinic N.^[^ ^37^ ^]^ The FT‐IR and XPS spectra confirmed the existence of hydroxyl group (─OH) and amino group (‐NH_2_) of CDs, which could bind to the inner PAA block of star‐liked di‐block copolymers β‐CD‐*g*‐PAA‐*b*‐POEGA to create ultra‐stable CDs assemblies through hydrogen bonding and electrostatic interactions.

To evaluate the optical performance of CDs, the UV–vis absorption, photoluminescent characteristics of the solution and powders were investigated. Figure 1c shows that absorption band of CDs solution has peaks at 218 and 420 nm, respectively, which are usually attributed to the n–π* conjugation and π–π transitions of C═C in the core of the CDs, respectively.^[^ ^38^, ^39^ ^]^ The CDs can be well‐dispersed in H_2_O and possess a maximum emission peak of 560 nm, and the optimal wavelength of excitation light was 470 nm (Figure 1c,d; Figure S12, Supporting Information).^[^ ^40^ ^]^ When the excitation wavelength changes from 320 to 500 nm, the corresponding emission wavelength shows two fluctuations and reached a maximum at the excitation wavelength of 470 nm. The CDs solution exhibits typical excitation‐independent fluorescence emission with a peak at 560 nm (Figure 1d; Figure S12, Supporting Information). It is noteworthy that the absorption band of the CDs powder appears only around 400 nm and no obvious characteristic peaks of fluorescence are observed (Figure S13, Supporting Information).

**Figure 2:**
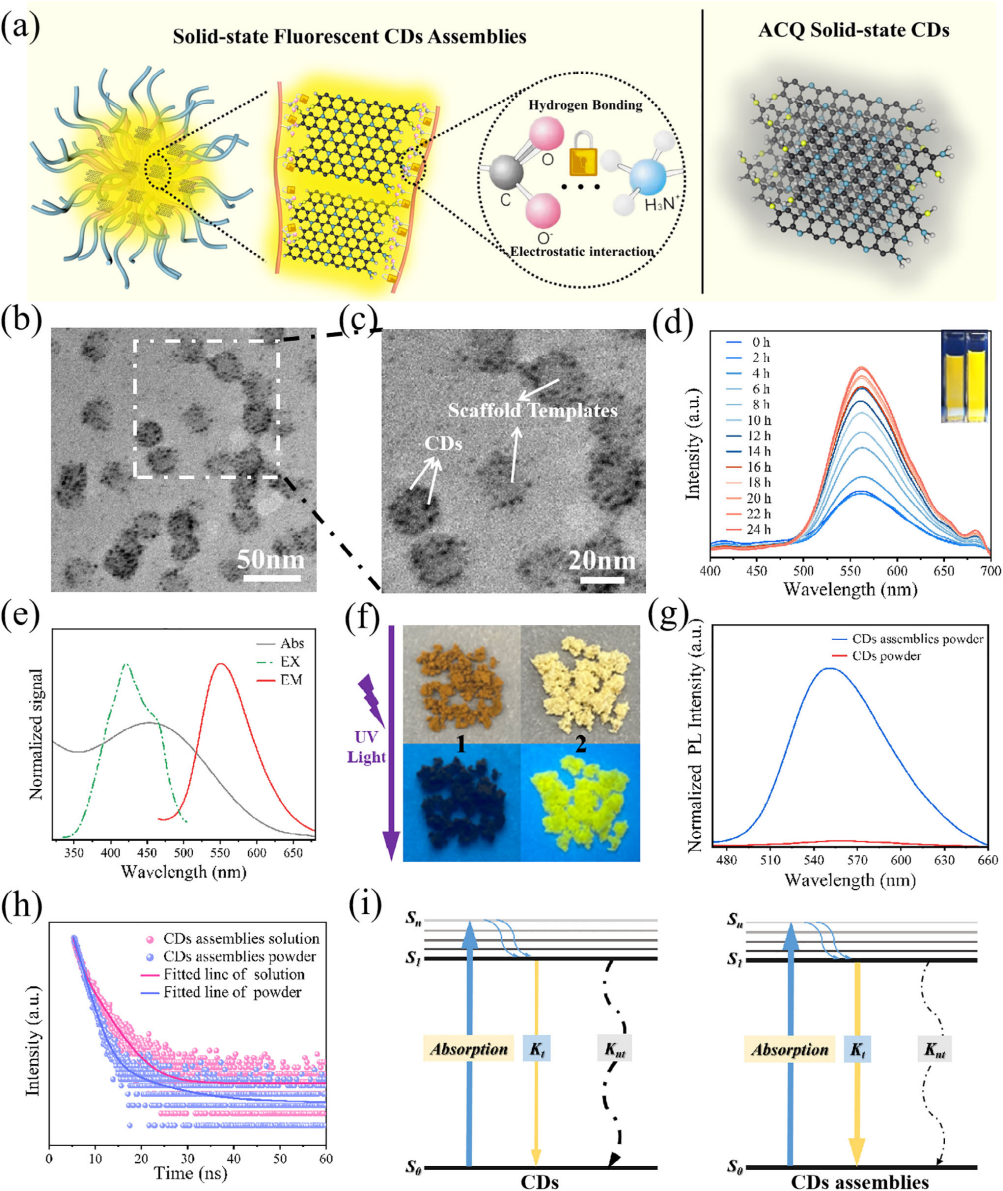
a) Schematic of hydrogen bonding and electrostatic interaction inside CDs and star‐like hydrophilic polymer; b,c) TEM image of CDs assemblies; d) Fluorescence absorption spectrum of fluorescent CDs assemblies over time, insets show digital photographs of CDs assemblies at 0 and 24 h in aqueous solution under UV light; e) UV−vis absorption, fluorescence emission spectra, and photoexcitation spectrum of CDs assemblies powder; f) Photographs of CDs (No. 1) and CDs assemblies (No. 2) powder under nature light (top) and UV light (bottom); g) Fluorescence emission spectra of CDs and CDs assemblies powder; h) Fluorescence decay curve of CDs assemblies powder and solution; i) Energy level diagram of effect of ACQ (left) in CDs powders and AIE (right) in CDs assemblies powders, respectively.

The absolute PLQY of CDs aqueous and powder was measured to be 1.81% and 0.12%, respectively. The time‐resolved PL spectrum showed that the average lifetime of CDs was 0.79 ns in aqueous solution and 1.04 ns in solid‐state under 470 nm irradiation (Figure 1e; Figure S14, Supporting Information). The CDs solution was emissive. However, the CDs powders were almost non‐emissive, this is a typical ACQ phenomenon (**Figure** **2**f). The concentration experiments also verified the ACQ characteristics of CDs, as their fluorescence emission intensity weakened with the increasing of CDs concentrations (Figure 1f,g; Figure S15, Supporting Information). These results indicated that when CDs were in solid‐state, they undergo ACQ effect owing to the π–π stacking interactions, which severely limit the application of CDs in high concentration or solid‐state. Interestingly, when CDs were dispersed in various ratios of water/glycerol mixtures, and as the proportion of the glycerol with high viscosity increases, the emission intensity continues to increase (Figure 1h). According to previous work, high‐viscosity media can effectively limit intramolecular interactions, suppressing non‐radiative decay channels, thereby resulting in enhanced luminescence intensity.^[^ ^41^, ^42^ ^]^ Thus, this phenomenon provides a feasible strategy to turn the ACQ CDs to solid‐state fluorescent CDs via the suppression of π–π stacking interactions and the vibration of CDs to avoid the non‐radiative decay ways.

To prepare the solid‐state fluorescent CDs assemblies, hydrophilic star‐liked polymers β‐CD‐*g*‐PAA‐*b*‐POEGA were used as scaffolds nanoreactors to encapsulate ACQ CDs via the polymer directed nanoconfined self‐assembly strategy. ─OH and ─NH_2_ on the surface of CDs interact with ─COOH group on PAA blocks through electrostatic interactions and hydrogen bonds, then CDs were encapsulated within the nanoconfined space of the inner PAA core. The outer POEGA block performed as a protective shell, endow the excellent hydrophilicity, stability and dispersibility to CDs assemblies (Figure 2a).^[^ ^29^, ^31^ ^]^ Intermolecular hydrogen bonds were formed by combining electron‐rich groups on ACQ CDs with electron‐rich groups on PAA blocks of star‐liked polymers. The FT‐IR spectra before and after the directed self‐assembly of CDs showed a significant shift in the infrared stretching vibration peak of the carboxyl group at 1635 cm^−1^, indicating presence of intermolecular hydrogen bonds in the CDs assemblies (Figure S16, Supporting Information). The strong attraction of hydrogen bonds increases the degree of conjugation and delocalization of the molecules, which allows the fluorescent CDs nanomaterials to firmly bind to the inner PAA block of the star‐liked polymer.^[^ ^43^ ^]^ As the CDs were confined into the inner core of the polymer micelles via the polymer directed nanoconfined self‐assembly process, CDs assemblies with stable and well‐defined superstructure can be prepared. TEM images of the CDs assemblies exhibit regular morphology and uniformly sized with diameters about 28 ± 4 nm, and the scaffolds template effectively prevents the direct contact between the individual CDs (Figure 2b,c). As the ACQ CDs could not contact with each other, the direct π–π stacking of the CDs were inhibited, thus, the ACQ phenomenon of solid‐state CDs were significantly suppressed. Furthermore, owing to the electrostatic interaction and hydrogen bonding between the CDs and the PAA block, the suppression of the vibration of the CDs greatly enhanced the emission intensity. As the inner P*t*BA polymer was prepared via photoinduced ATRP, the molecular weight distribution was very low (<1.25), thus, the PAA core and the CDs assemblies were nearly monodispersed and possess regular morphology (Figure 2b,c).

To gain insight into the basic luminescent properties of CDs assemblies, the absolute PLQY and emission decay profiles were measured. The absolute PLQY of the CDs assemblies was 8.90% in aqueous solution, significantly lower than that (21.46%) in solid‐state. This phenomenon illustrates a representative example of aggregation induced emission. Time‐resolved PL spectroscopy showed that the average lifetimes of CD assemblies are 1.23 ns in aqueous solution and 1.83 ns in solid state when excited at 365 nm (Figure 2h). To evaluate and confirm the AIE effect, the radiative rate constant (*K*_r_) and non‐radiative rate constant (*K*_nr_) of CDs powder and CDs assemblies powder were calculated according to the following formula:^[^ ^44^ ^]^

(1) Kr=PLQY/τ

(2) Knr=(1−PLQY)/τ

**Table** **1** shows that *K*_r_ values for the solid‐state CDs assemblies are relatively high, with corresponding low *K*_nr_ values, consistent with the restriction of intramolecular motion (RIM) mechanism.^[^ ^45^, ^46^ ^]^ In the aggregation state, the interaction of surrounding molecules restricts the vibration and rotation of AIE intramolecular bonds, thus inhibiting non‐radiative decay and resulting in enhanced fluorescent intensity.^[^ ^41^ ^]^ Therefore, we speculate that the polymer directed nanoconfined self‐assembly of ACQ CDs inside of star‐liked polymer nanoconfined space inhibits the movement of surface groups of CDs and the π–π stacking interactions of CDs in the assemblies, thus, resulting in significant reduction in the non‐radiative rate, avoiding the ACQ phenomenon, thereby improving the PL efficiency of CDs assemblies (Figure 2a,i).

**Table 1:** PLQY, *τ*, *K*_r_, *K*_nr_ of CDs and CDs assemblies in solid‐state.

Entry	PLQY[%]	Τ[ns]	K_r_[ns^−1^]	K_nr_[ns^−1^]
CDs powders	0.12	1.04	0.0012	0.9604
CDs assemblies powders	21.46	1.83	0.1173	0.8827

Compared with bare CDs, the fluorescence spectrum of CDs assemblies exhibits no obvious shift, and the emission band remains unchanged across different excitation wavelength, which is inherited by CDs (Figure 2e). The fluorescence of solid‐state CDs assemblies and bare CDs exposed to natural and UV light were compared. It is quite apparent that the fluorescence intensity of the solid‐state CDs assemblies was significantly higher than bare solid‐state CDs under UV light (Figure 2f,g). Figures S18 and S19 (Supporting Information) depict the PL spectra of assemblies solution and powder at various excitation wavelengths. When the excitation wavelength changed from 320 to 500 nm, the emission peak predominantly appears at 560 nm and remained relatively unchanged. The emission intensity of assemblies powder and solution both reached highest when the excitation wavelength was 420 nm. The above results indicate that the CDs assemblies not only exhibits excellent solid‐state fluorescent properties, which arise from the suppression of π–π stacking and the RIM mechanism, but also retains the original fluorescent characteristics of the individual CDs. It is worth noting that the solid‐state fluorescence and aggregation induced emission enhancement phenomenon of CDs assemblies in this system originates from the spatial confinement effect and the restricted intramolecular motion of CDs by the star‐liked polymer template rather than the intrinsic AIE properties of CDs, which provides a novel strategy for expanding the solid‐state luminescence applications of other ACQ materials.

In order to investigate the nanoconfined self‐assembly process and the variation of fluorescence emission characteristics of CDs assemblies, the evolution of fluorescence intensity was dynamically monitored. The fluorescence intensity of CDs assemblies gradually increased as the assembly time advanced, ultimately reaching the highest value after 22 h. This indicates that the assembly process was completed and the PAA polymer was fully integrated with the CDs functional group (Figure 2d; Figure S17, Supporting Information). The fluorescent intensity increased with the assembly time was ascribed to the gradual aggregation of CDs inside of the nanoconfined polymer template. The aggregation of ACQ CDs resulted in the aggregation induced emission enhancement effect, which is likely due to the restricted mobility of CDs and the π–π stacking interactions between them.^[^ ^47^ ^]^

**Figure 3:**
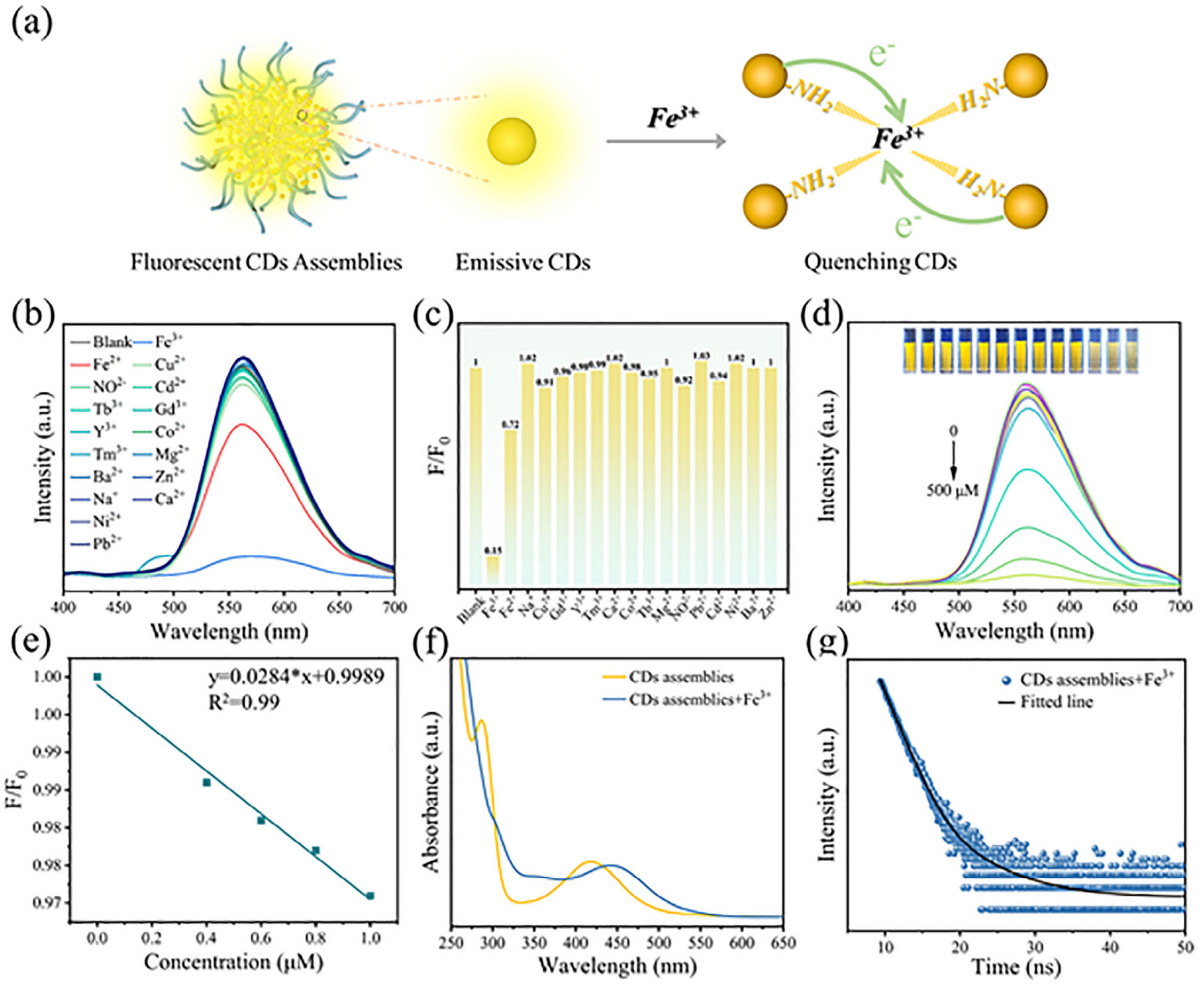
a) Fe^3+^ ion specific detection mechanism of the CDs assemblies; b) PL spectra of CDs assemblies detecting different metal ions; c) Histogram of the relative emission intensity of CDs assemblies detecting different metal ions; d) PL spectra of CDs assemblies with different Fe^3+^ concentrations, inset showed the optical photographs under UV light; e) Relationship between fluorescence intensity and Fe^3+^ concentration (0–1 µm); f) UV–vis spectrum of CDs assemblies in the presence and absence of Fe^3+^; g) Fluorescence decay curve of CDs assemblies in the presence of Fe^3+^.

The bare CDs usually suffer from structural damage or random aggregation of nanostructures due to the spontaneous decrease in surface energy, leading to poor fluorescence stability (Figure S21a, Supporting Information). In contrast, the polymer template directly nanoconfined self‐assembly strategy can efficiently and accurately prepare CDs assemblies with regular spherical morphology and thermodynamic stability. After storage for one month, the fluorescence intensity remained basically unchanged and no aggregation was observed (Figure S21b, Supporting Information). The fluorescence stability of the assemblies in different acid‐base conditions and temperatures were also examined. As depicted in **Figure** **3**a–d, the assemblies exhibited robust fluorescence stability under a broad range of environmental conditions (T = 20–100 °C, pH = 4–11). However, under extreme pH conditions (pH < 3 and pH > 12), the fluorescence intensity of the system decreased. To better investigate the effect of pH on the luminescence and stability of the assemblies, the relationship between zeta potential and pH values were explored. As shown in Figure S23 (Supporting Information), the relationship between the zeta potential and pH can be described by a fitted quadratic equation: *y* = 0.99x^2^−20.43*x* + 35.88, indicating the reversible protonation/deprotonation of functional groups in the system. From the equation, the calculated isoelectric point (pI) of the CDs assemblies is 1.94. In acidic environment, the system gradually collapses due to the protonation of the PAA block. However, the carboxyl group (‐COOH) of the PAA block was not fully protonated and the amino group (‐NH_3_^+^) on the surface of the CDs can always form a charge neutralization interface through electrostatic bonding. As the pH approaches and lower than the isoelectric point (pI = 1.94), CDs assemblies become unstable and prone to agglomeration due to the increasing electrostatic repulsion of the system,^[^ ^48^ ^]^ resulting in the fluorescence weakens under the condition of pH = 3. Under alkaline conditions, the deprotonation of the ‐COOH increases the electrostatic attraction of the system, and the – NH_3_^+^ on the surface of the CDs also form hydrogen bond with OH^−^. Under extremely alkaline conditions, – NH_3_^+^ on the surface of the CDs changed to ‐NH_2_ without positive charge, and the carboxyl group of PAA will become ‐COO^−^, thus, CDs will separate with PAA that will result in the sudden decrease of the luminescent intensity.^[^ ^49^ ^]^ Simultaneously, POEGA polymer chains on the exterior is permanently covalent attachment to the CDs assemblies core, providing spatial protection that reduces direct contact between the CDs and the external environment, thereby preventing pH‐driven aggregation or surface reactions.^[^ ^50^ ^]^

The CDs assemblies were synthesized using star‐liked polymer templates of varying molecular weights, with a fixed mass ratio of ‐COOH to CDs maintained across all templates. Interestingly, the fluorescence intensity of the CDs assemblies increased with the increasing of molecular weight of polymer template (Figure 3e,f). As the CDs mainly combine with PAA block, thus, the size of PAA will represent the size of CDs assemblies. To explain this phenomenon, particle size analysis of star‐liked PAA polymers was performed. As shown in Figure S24 (Supporting Information), the hydrodynamic diameter of the hydrolyzed star‐liked PAA polymers gradually increased from 19.8 to 172.8 nm as the molecular weight of the star‐liked P*t*BA polymers increased. This indicates that at identical polymer template content, higher molecular weight polymer templates possess more extensive PAA blocks that can accommodate greater quantities of CDs, then more CDs continuously aggregate inside of template, resulting in increased PL intensity, which is a typical AIE phenomenon. Subsequently, different concentrations of templates and the same amounts of CDs were employed for the self‐assembly. As depicted in Figure S25 (Supporting Information), the fluorescence intensity increased with the decrease of the concentration of the template after assembly for 24 h, suggesting that at lower template concentration, each unimolecular micelle could adequately combine with CDs, thus encapsulating more CDs and exhibiting enhanced fluorescence intensity.

**Figure 4:**
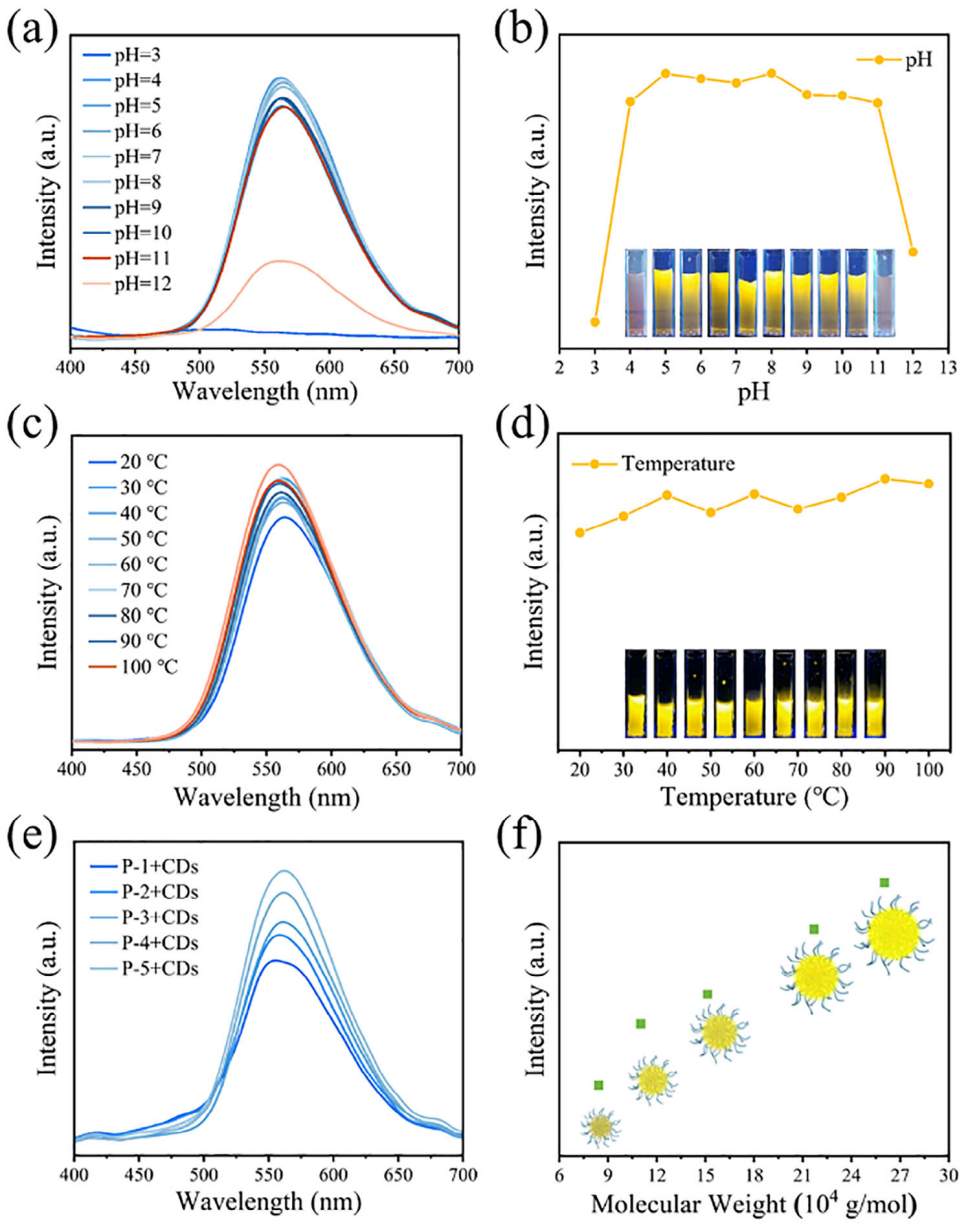
a) PL spectra of CDs assemblies at different pH; b) Trend of emission peak of fluorescent CDs assemblies at different pH values (*λ*_em_ = 560 nm); c) PL spectra of CDs assemblies at different temperature; d) Trend of emission peak of fluorescent CDs assemblies at different temperature (*λ*_em_ = 560 nm); e) PL spectra of CDs assemblies with different molecular weights; f) Relationship between the fluorescence intensity of the CDs assemblies and the molecular weight of the inner block P*t*BA polymers.

The prepared CDs assemblies possessed strong specificity for Fe^3+^ ions, enabling qualitative detection of Fe^3+^ (**Figure** **4**b,c). To further determine the sensitivity of CDs assemblies to Fe^3+^ detection, the PL spectra of CDs assemblies were collected at different concentration of Fe^3+^ ions. The fluorescent intensity of CDs assemblies gradually decreases with increasing concentrations of Fe^3+^ ions (Figure 4d). Figure 4e illustrates the fluorescence intensity versus Fe^3+^ ion concentration, and the fitting results showed that within 0–1 µm range, the PL intensity ratio (F/F_0_) had a significant linear correlation with Fe^3+^ concentration (*R* ^2^ = 0.99). The detection limit is 0.127 µm, indicating efficient detection ability CDs assemblies for Fe^3+^ ions. Compared with most of the reported fluorescent Fe^3+^ ions probes, the CDs assemblies for Fe^3+^ ions detective sensor showed better specificity and stability (Table S4, Supporting Information). Fe^3+^ ions have a strong affinity interaction with ‐NH_2_ and ‐OH of CDs, leading to the aggregation of CDs, which is the reason for the specifically quenching of CDs by Fe^3+^ ions. Irregular aggregated CDs promoted the complexation of non‐radiative holes and electrons, and the excited electrons of CDs will transfer to Fe^3+^ ions, resulting in fluorescence quenching. Previous studies have shown that fluorescence quenching induced by CDs aggregation is static quenching.^[^ ^51^ ^]^ To elucidate the mechanism of fluorescence quenching, the UV–vis absorption and fluorescence lifetime of CDs assemblies before and after Fe^3+^ addition were analyzed. As illustrated in Figure 4f,g, the UV‐vis spectrum of the CDs assemblies showed a slight redshift after Fe^3+^ addition, and the PL lifetime was 1.70 ns, there is no significant change compared to 1.83 ns in the absence of Fe^3+^ (Figure 2h), indicating that the quenching process is static quenching.

**Figure 5:**
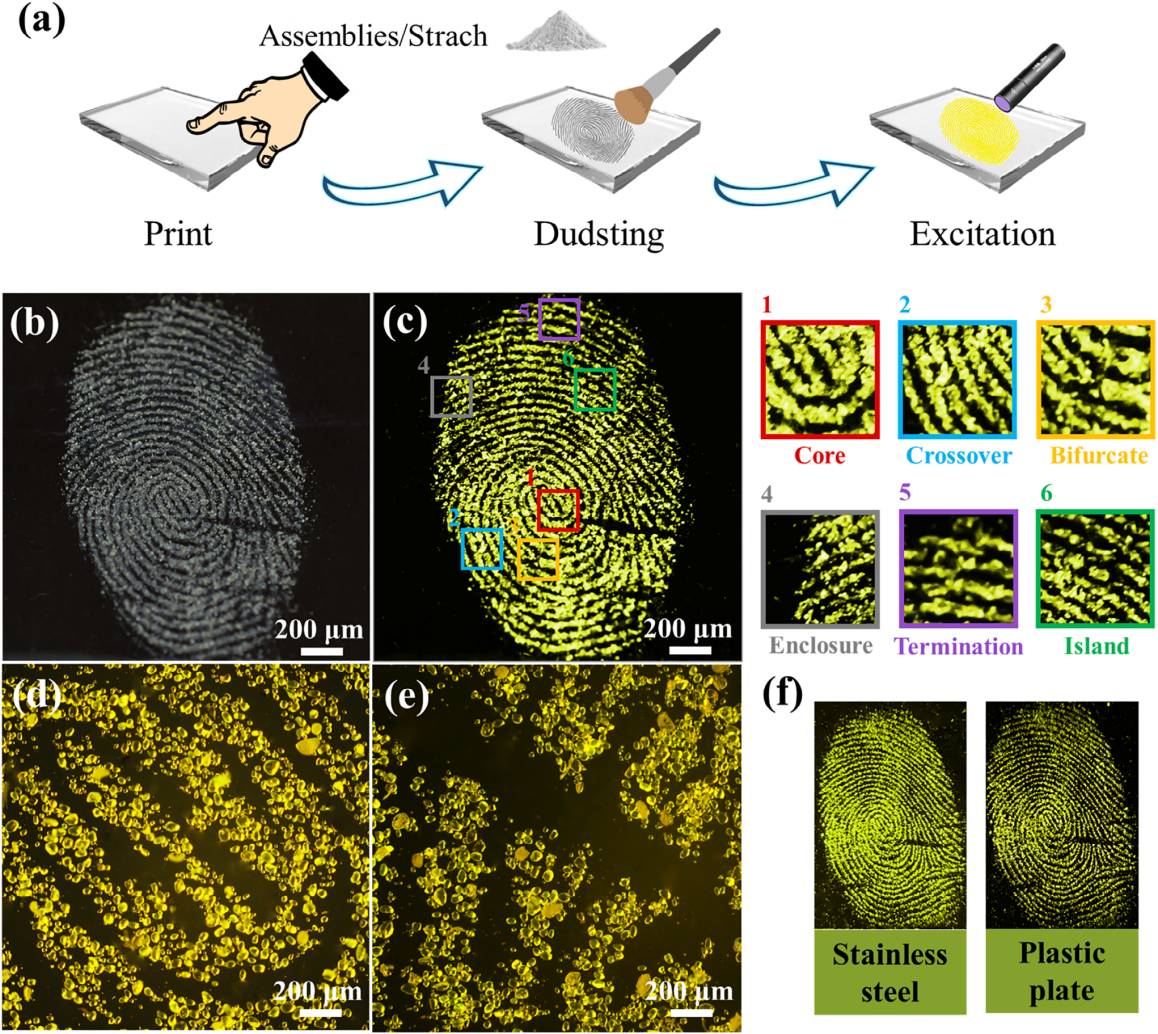
a) Schematic of instant visualization of latent fingerprints; Photographs of CDs assemblies/starch LFPs under b) sunlight and c) UV light, magnified photos of (c): c1) core, c2) crossover, c3) bifurcate, c4) enclosure, c5) termination, c6) island; d) Fluorescence images of CDs assemblies/starch fingerprints, and e) same sample after one month storage; f) Fluorescent images of LFPs on different substrate.

As previously mentioned, the prepared CDs assemblies has strong yellow fluorescence in solid‐state, nanoscale and uniform size, and overcomes the ACQ dilemma, rendering them potential phosphors for latent fingerprints (LFP) high‐sensitivity imaging.^[^ ^52^, ^53^ ^]^ By employing a powder mixture of CDs assemblies and starch, LFP on glass was extracted via powder dusting (**Figure** **5**a). As illustrated in Figure 5b,c, under sunlight and UV light illumination, the powder mixture adhered to the glass slide reveal clear fingerprint image ridges, the high‐contrast fluorescent signal makes major features such as arches, loops and spirals clearly visible. The magnified region images clearly revealed the minor features, including the (c1) core, (c2) crossover, (c3) bifurcate, (c4) enclosure, (c5) termination, and (c6) island, which is the most important identifying information in analysis of fingerprint.^[^ ^54^ ^]^ The fluorescent image of the fingerprint on the glass distinctly show the yellow‐emitting CDs assemblies/starch mixture, which attached to the finger residue, accurately showing the details of this fingerprint. After storage for one month, the fluorescent figure of the fingerprint was still bright and clear. To demonstrate the wide applicability of CDs assemblies, we also selected stainless steel and plastic plate as substrates, the CDs assemblies showed excellent ridge details on these substrates (Figure 5f). Owing to the unique properties of CDs assemblies, it can be machined into different forms to suit various applications. Figure S31 (Supporting Information) showed a flexible film with yellow light‐emitting prepared by CDs assemblies. In this case, polyvinyl alcohol (PVA) was employed as a film substrate because of high transmittance. The CDs assemblies/PVA films were transparent under natural light and emitted a strong yellow luminescent under UV light, suggesting that the CDs assemblies powder have potential as dopants for organic light‐emitting diodes (OLED).

**Figure 6:**
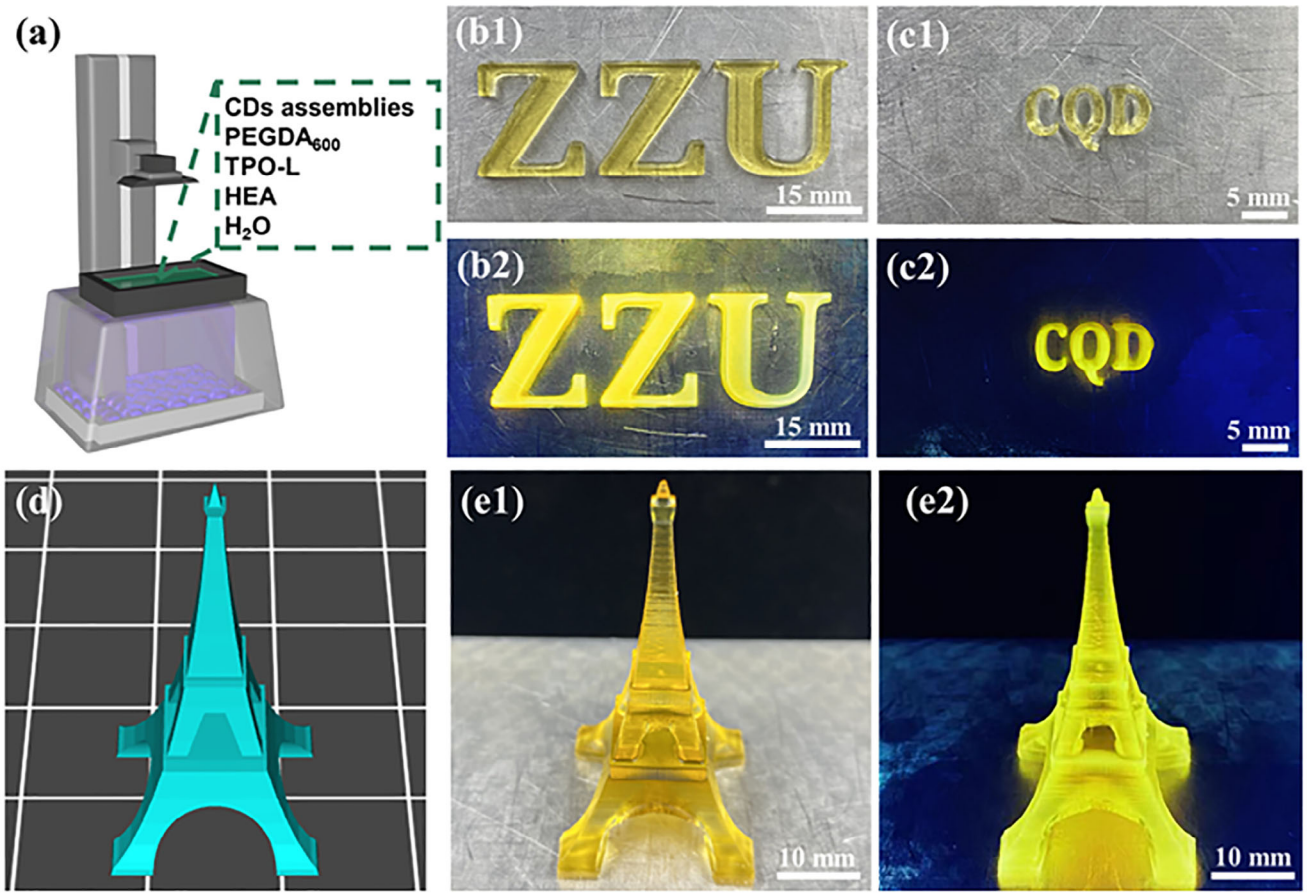
a) Schematic of 3D printing with CDs assemblies as luminescent materials; Optical photographs of “ZZU” and “CQD” prints under sunlight (b1, c1) and UV light (b2, c2); d) Models of the simple the Eiffel Tower; Optical photographs of the Eiffel Tower prints under sunlight (e1) and UV light (e2).

In addition, CDs assemblies can also be used for digital light processing (DLP) 3D printing (**Figure** **6**a). Figure 6b,c shows digital photos of printed objects with different sizes under natural and UV light, respectively. The excellent optical characteristics of CDs assemblies provide well photoluminescent properties for prints. Under UV light, the printed objects show strong yellow luminescent (Figure 6b2–c2), suggesting that during the 3D printing process, CDs assemblies, as solid‐state light‐emitting elements in raw materials of 3D printing, were uniformly embedded into the polymer matrix. The emission intensity of the printed “AIE” with CDs assemblies was higher than the printed “ACQ” with CDs. Like most organic dyes, ACQ CDs will greatly reduce their intensity after exposure for 1 min under UV light,^[^ ^55^ ^]^ while CDs assemblies show good photostability, under continuous UV irradiation for 30 min, the luminescence intensity of the printed hydrogel did not change significantly (Figure S32, Supporting Information). Figure 6d is a more complex Eiffel Tower model. It can be seen from Figure 6e that the object was successfully printed and showed excellent print resolution. Similarly, the printed Eiffel Tower showed intense yellow fluorescence under UV light, indicating that functional hydrogel with fluorescence was successfully prepared by DLP 3D printing.

## 3 Conclusion

In conclusion, by utilizing the hydrophilic star‐liked polymer β‐CD‐*g*‐PAA‐*b*‐POEGA unimolecular micelles as a nanoconfined nanoreactor, the ACQ CDs was successfully transformed to AIE CD assemblies with solid‐state fluorescence, regular spherical structures, uniform size, and enhanced structural stability. The size and fluorescence intensity of these assemblies can be precisely controlled through the photoinduced ATRP process. Importantly, the scaffold template prevents π–π stacking interactions between the surface groups of CDs and suppress the vibration of CDs, thereby inhibiting ACQ and reducing non‐radiative decay, thus effectively facilitates the transition from ACQ to AIE, enabling the solid‐state luminescence of the CD assemblies. The successful fabrication of these assemblies highlights the efficiency of the polymer direct nanoconfined self‐assembly strategy and expands the potential for their practical applications. These assemblies show promise as solid‐state luminescent materials for the development of real‐time visualization of latent fingerprints, flexible films, and 3D functional hydrogels, offering excellent fluorescence properties for a wide range of applications. This study not only deepens our understanding of the AIE mechanism in the aggregation of nanomaterials but also demonstrates an effective strategy for constructing ultra‐stable, tunable fluorescent materials, which is of great significance for advanced detection technologies and materials science, and also lays the foundation for future research on the controlled assembly of other ACQ nanomaterials for solid‐state fluorescence applications.

## 4 Experimental Section

### Materials

β‐cyclodextrin (β‐CD, Sigma–Aldrich) was used as received. 2‐Bromoisobutyryl bromide (98%), o‐phenylenediamine (99%), *tert*‐butyl acrylate (*t*BA, 99%), tris(2‐dimethylaminoethyl)amine (Me_6_TREN, 99%), and copper bromide (98%), oligo(ethylene glycol) acrylate (OEGA, *M*_n_ = 480 g mol^−1^) were purchased from Aladin Chemical Reagent Company and used as received. OEGA and *t*BA passed over alumina columns before use. All other reagents were purified by ordinary purification procedures.

### Synthesis of Heptakis [2,3,6‐tri‐O‐(2‐Bromo‐2‐Methylpropionyl)]‐β‐Cyclodextrin) (21‐Br‐β‐CD)

β‐CD (6.82 g) was pre‐dried in a vacuum oven at 60 °C for 24 h to remove moisture. The dried β‐CD was then dissolved in 60 mL anhydrous 1‐methyl‐2‐pyrrolidione (NMP) and cooled to 0 °C in the ice‐water bath with magnetic stirring. 2‐bromoisobutyryl bromide (58.0 mL) was added dropwise to the reaction system over 2 h. After the addition, the mixture was kept at 0 °C and stirred magnetically for an additional 2 h. After the reaction system reached room temperature, continue magnetic stirring for 24 h. The resulting brown solution was diluted with 150 mL of dichloromethane, sequentially extracted three times with saturated NaHCO_3_ aqueous solution and deionized water. The organic phase was separated and concentrated under reduced pressure using a rotary evaporator. The concentrated product was then added dropwise to 500 mL of ice‐cold hexane for precipitation. The precipitate was collected by filtration and dried in a vacuum oven at 40 °C for 24 h to yield the functional initiator 21‐Br‐β‐CD.

### Synthesis of Fluorescent Carbon Dots(CDs)

O‐phenylenediamine (1.0 g) was dissolved in 100 mL of deionized water and stirred magnetically until fully dissolved. The solution was then transferred to a 100 mL Teflon hydrothermal reactor. Once the drying oven is fully preheated to the set temperature of 200 °C, place the hydrothermal reactor into the drying oven for a continuous hydrothermal reaction for 8 h. After the hydrothermal reaction was finished, turn off the heating switch of the drying oven allow it to cool naturally to room temperature, and obtain the product solution. Then use a dialysis membrane with a pore size of 1000 Da to perform dialysis filtration for 24 h. After the dialysis was completed, the residual solution in the dialysis bag was collected and freeze‐dried for 24 h to obtain yellow fluorescent carbon dots (CDs).

### Synthesis of 21‐Arm, Star‐Like β‐CD‐g‐PtBA by ATRP Using 21‐Br‐β‐CD as Macroinitiators

The polymerization of *t*BA was carried out using 21‐Br‐β‐CD as a macroinitiator. 10 mg of 21 Br‐β‐CD (1 equiv. of Br in 21Br‐β‐CD), 4.3 mL of *t*BA (600 equiv.), 0.23 mg of CuBr_2_ (0.01 equiv.), 5.23 mg of Me_6_TREN (0.04 equiv.), and 4.3 mL of DMF were added to a 10 mL Schlenk flask. The solution was bubbled with nitrogen for 1 h to fully remove oxygen, subsequently the sealed reaction vials were placed in a 365 nm UV light reactor for polymerization. At the same time, the external fan was used to cool down the reaction device. After the prescribed time, the flask was removed to allow air to enter and placed in liquid nitrogen to terminate the reaction. The raw product was then diluted with THF, passed through a neutral alumina column to remove the catalyst. The polymer solution was precipitated three times in precipitant (ice methanol: deionized water = 1:1, v/v). After purification, the precipitate was dried in vacuum at 40 °C for 24 h to obtain star‐like β‐CD‐*g*‐P*t*BA.

### Synthesis of 21‐Arm, Star‐Like β‐CD‐g‐PtBA‐b‐POEGA by ATRP Using 21‐Arm, Star‐Like PtBA as Macroinitiators

The polymerization of OEGA was carried out using star‐like β‐CD‐*g*‐P*t*BA as macroinitiator. β‐CD‐*g*‐P*t*BA (0.01 mmol, 1 equiv.), OEGA (0.6 mmol, 60 equiv.), CuBr_2_ (0.1 µmol, 0.01 equiv.), Me_6_TREN (0.4 µmol, 0.04 equiv.), and DMF (DMF: OEGA = 1:1, v/v) were added to a 10 mL Schlenk flask. The solution was bubbled with nitrogen for 1 h to fully remove oxygen, subsequently the sealed reaction vials were placed in a 365 nm UV light reactor for polymerization. At the same time, the external fan was used to cool down the reaction device. After a period of polymerization, the flask was removed to allow air to enter and placed in liquid nitrogen to terminate the reaction. The mixture was then diluted with THF and passed through a neutral alumina column to remove the copper salts. The polymer was precipitated with an excess of cold n‐hexane, the precipitate was dried in vacuum at 40 °C for 24 h to obtain star‐like β‐CD‐*g*‐P*t*BA‐*b*‐POEGA.

### Synthesis of 21‐Arm, Star‐Like β‐CD‐g‐PAA‐b‐POEGA

The star‐like β‐CD‐*g*‐P*t*BA‐*b*‐POEGA (0.3 g) was dissolved in CH_2_Cl_2_ solvent (30 mL), and then trifluoroacetic acid (10 mL) was slowly added to the above system to hydrolyze the P*t*BA block into polyacrylic acid (PAA) block. The crude product β‐CD‐*g*‐PAA‐*b*‐POEGA obtained by hydrolysis was concentrated by rotary evaporator and diluted with THF. Subsequently, the polymer solution was precipitated by cold n‐hexane three times, the precipitate was collected and freeze‐dried for 24 h to obtain β‐CD‐*g*‐PAA‐*b*‐POEGA.

### Synthesis of 21‐Arm, Star‐Like β‐CD‐g‐PAA

The star‐like β‐CD‐*g*‐P*t*BA (0.3 g) was dissolved in CH_2_Cl_2_ solvent (30 mL), and then trifluoroacetic acid (10 mL) was slowly added to the above system to hydrolyze the P*t*BA block into polyacrylic acid (PAA) block. The crude product β‐CD‐*g*‐PAA obtained by hydrolysis was concentrated by rotary evaporator and diluted with THF. Subsequently, the polymer solution was precipitated by cold n‐hexane three times, the precipitate was collected and freeze‐dried for 24 h to obtain β‐CD‐*g*‐PAA.

### Polymer Micelles Directed Self‐Assembly of CDs Assemblies

The CDs (0.5 mg mL^−1^, 0.5 mL) were mixed with β‐CD‐*g*‐PAA‐*b*‐POEGA (1 mg mL^−1^, 0.5 mL) in deionized water. The solution was mixed and allowed to stand for 24 h to obtain homogeneously assembled solution of CDs assemblies. The obtained solution freeze‐dried for 24 h to obtain CDs assembly powder.

### Development and Imaging of Latent Fingerprints (LFPs)

In this study, all fingerprints were collected from the right thumb of male donors. Prior to contacting various substrates, hands were washed with soap and water, followed by gently wiping them on the forehead. Fingers were then pressed onto the substrate, and powder was subsequently sprayed onto the surface, with excess powder brushed away using a small brush. Finally, under 365 nm UV light exposure, yellow fingerprint impressions became clearly visible. The substrates utilized included glass slides, plastic sheets, and stainless steel.

### DLP 3D Printing

A typical procedure for fabricating 3D printed objects is as follows: A 20 mL glass vial was added HEA (5.5 g), followed by PEGDA_600_ (1.66 g), TPO (0.03 g), CDs assemblies (0.2 g), and deionized water (5 mL). Printing parameters concluding Z lift speed and Z retract speed were set to 0.50 mm s^−1^, and the Z lift distance was set to 3.00 mm, respectively. The regular cure time per layer (0.2 mm) was 15 s. DLP 3D printer (Anycubic Photon Ultra) with a violet (λmax = 405 nm) light LED array.

### Fabrication of Yellow‐Light‐Emitting Film

PVA (1 g) was dissolved in CDs assemblies aqueous solution (20 mL) following sonication for 30 min. The transparent CDs assemblies/PVA film were finally prepared by molding at 60 °C.

## Conflict of Interest

The authors declare no conflict of interest.

## Supporting information



Supporting Information

## Data Availability

The data that support the findings of this study are available from the corresponding author upon reasonable request.
