# Supplementary material for: Precision Nanoconfined Self‐Assembly of ACQ Carbon Dots for Enhanced Solid‐State Fluorescence
Source: Adv Sci (Weinh). 2025 May 8;12(27):2503317. doi: 10.1002/advs.202503317 (PMC12279204; doi:10.1002/advs.202503317)
Supplement: Supplementary file 1 — Supporting Information [file ADVS-12-2503317-s001.pdf]

ADVANCED SCIENCE
Open Access

# Supporting Information

for *Adv. Sci.*, DOI 10.1002/advs.202503317

Precision Nanoconfined Self-Assembly of ACQ Carbon Dots for Enhanced Solid-State Fluorescence

*Jingyi Hao, Wenjie Zhang*, Yuying Li, Wenjun Ma, Yueying Zhu, Junle Zhang, Ge Shi, Xiaoguang Qiao, Yanjie He, Zheng Zhao*, Xinchang Pang* and Ben Zhong Tang**

# Supporting Information

## Precision Nanoconfined Self-Assembly of ACQ Carbon Dots for Enhanced Solid-State Fluorescence

Jingyi Hao,[a]+ Wenjie Zhang, [a]+* Yuying Li,[a] Wenjun Ma,[a] Yueying Zhu,[d] Junle Zhang,[a] Ge Shi,[a] Xiaoguang Qiao,[a] Yanjie He,[a] Zheng Zhao,[b]* Xinchang Pang,[a] [d]* Ben Zhong Tang[b] [c]*

[+] These authors contributed equally to this work.

[a] J. Hao,+ Dr. W. Zhang,+ Y. Li, W. Ma, Dr. J. Zhang, Prof. G. Shi, Prof. X. Qiao, Prof. Y. He, Prof. X. Pang
Henan Joint International Research Laboratory of Living Polymerizations and Functional Nanomaterials, Henan Key Laboratory of Advanced Nylon Materials and Application, School of Materials Science and Engineering
Zhengzhou University
Zhengzhou, 450001, P. R. China
E-mail: zzuzwj2021@163.com
pangxinchang1980@163.com

[b] Prof. Z. Zhao, Prof. B. Tang
School of Science and Engineering, Shenzhen Institute of Aggregate Science and Technology, Shenzhen Key Laboratory of Functional Aggregate Materials
The Chinese University of Hong Kong
Shenzhen, Guangdong, 518172, China
E-mail: zhaozheng@cuhk.edu.cn
tangbenz@cuhk.edu.cn

[c] Prof. B. Tang
Department of Chemistry and the Hong Kong Branch of Chinese National Engineering Research Center for Tissue Restoration and Reconstruction
The Hong Kong University of Science and Technology
Clear Water Bay, Kowloon, Hong Kong, 999077, China

[d] Dr. Y. Zhu, Prof. X. Pang
School of Materials Science and Engineering, School of Chemistry & Chemical Engineering
Henan University of Science and Technology
Luoyang 471023, PR China

# Experimental Section

## Chemicals

β-cyclodextrin (β-CD, Sigma-Aldrich) was used as received. 2-Bromoisobutyryl bromide (98%), tris(2-dimethylaminoethyl)amine ($Me_6$TREN, 99%), and copper bromide (98%), oligo(ethylene glycol) acrylate (OEGA, $M_n$=480 g/mol) were purchased from Aladin Chemical Reagent Company and used as received. *Tert*-butyl acrylate (*t*BA), oligo (ethylene glycol) acrylate (OEGA) passed over alumina columns before use. All other reagents were purified by ordinary purification procedures.

## Synthesis of Heptakis [2,3,6-tri-O-(2-bromo-2-methylpropionyl)]-β-cyclodextrin) (21-Br-β-CD)

β-CD (6.82 g) was pre-dried in a vacuum oven at 60 °C for 24 h to remove moisture. The dried β-CD was then dissolved in 60 mL anhydrous 1-methyl-2-pyrrolidione (NMP) and cooled to 0 °C in the ice-water bath with magnetic stirring. 2-bromoisobutyryl bromide (58.0 mL) was added dropwise to the reaction system over 2 h. After the addition, the mixture was kept at 0 °C and stirred magnetically for an additional 2 h. After the reaction system reached room temperature, continue magnetic stirring for 24 h. The resulting brown solution was diluted with 150 mL of dichloromethane, sequentially extracted three times with saturated $NaHCO_3$ aqueous solution and deionized water. The organic phase was separated and concentrated under reduced pressure using a rotary evaporator. The concentrated product was then added dropwise to 500 mL of ice-cold hexane for precipitation. The precipitate was collected by

filtration and dried in a vacuum oven at 40 °C for 24 h to yield the functional initiator 21-Br-β-CD.

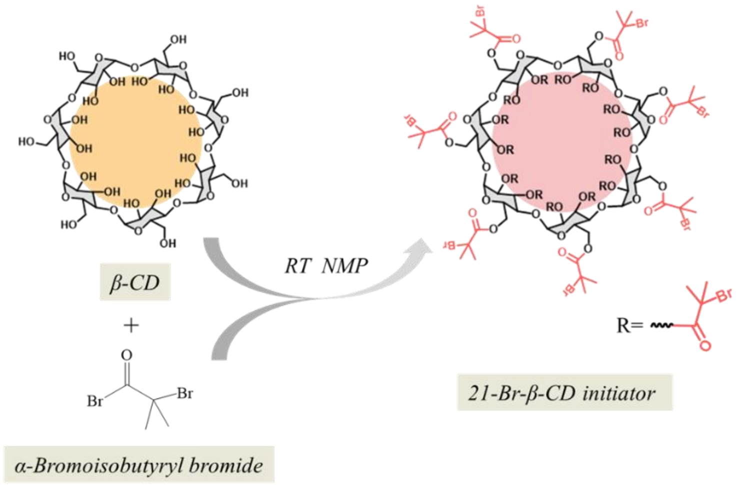


**Scheme S1**. Synthetic route for 21-Br-β-CD.

**Synthesis of fluorescent carbon dots（CDs）**

O-phenylenediamine (1.0 g) was dissolved in 100 mL of deionized water and stirred magnetically until fully dissolved. The solution was then transferred to a 100 mL Teflon hydrothermal reactor. Once the drying oven is fully preheated to the set temperature of 200 °C, place the hydrothermal reactor into the drying oven for a continuous hydrothermal reaction for 8 h. After the hydrothermal reaction was finished, turn off the heating switch of the drying oven allow it to cool naturally to room temperature, and obtain the product solution. Then use a dialysis membrane with a pore size of 1000 Da to perform dialysis filtration for 24 h. After the dialysis was completed, the residual solution in the dialysis bag was collected and freeze-dried for 24 h to obtain yellow fluorescent carbon dots (CDs)

**Synthesis of 21-Arm, Star-Like β-CD-*g*-P*t*BA by ATRP Using 21-Br-β-CD as**

## Macroinitiators

The polymerization of *t*BA was carried out using 21-Br-β-CD as a macroinitiator. 10 mg of 21 Br-β-CD (1 equiv. of Br in 21Br-β-CD), 4.3 mL of *t*BA (600 equiv.), 0.23 mg of $CuBr_2$ (0.01 equiv.), 5.23 mg of $Me_6$TREN (0.04 equiv.), and 4.3 mL of DMF were added to a 10 mL Schlenk flask. The solution was bubbled with nitrogen for 1 h to fully remove oxygen, subsequently the sealed reaction vials were placed in a 365 nm UV light reactor for polymerization. At the same time, the external fan was used to cool down the reaction device. After the prescribed time, the flask was removed to allow air to enter and placed in liquid nitrogen to terminate the reaction. The raw product was then diluted with THF, passed through a neutral alumina column to remove the catalyst. The polymer solution was precipitated three times in precipitant (ice methanol: deionized water =1:1, v/v). After purification, the precipitate was dried in vacuum at 40 °C for 24 h to obtain star-like β-CD-*g*-P*t*BA.

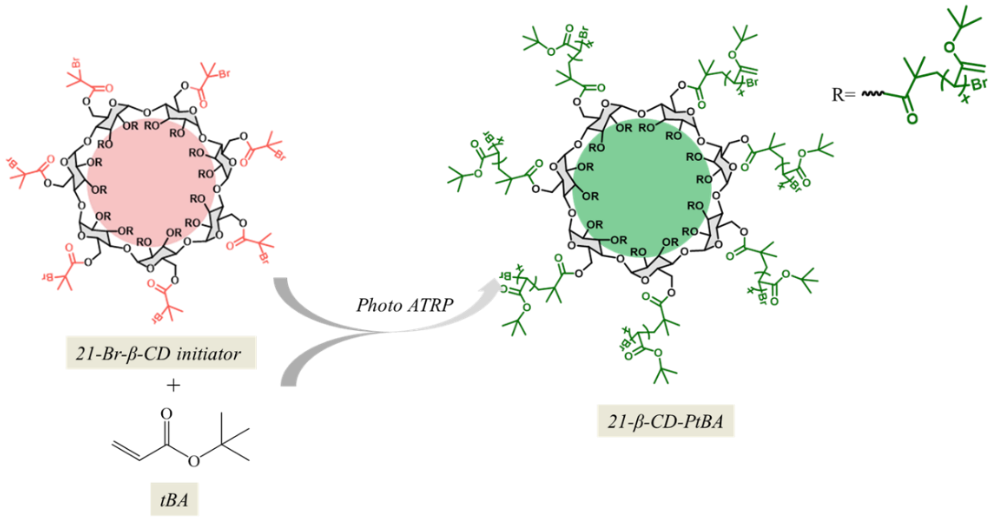


**Scheme S2**. Synthetic route for 21-Br-β-CD-P*t*BA.

**Synthesis of 21-Arm, Star-Like β-CD-*g*-P*t*BA-b-POEGA by ATRP Using 21-Arm,**

**Star-Like P*t*BA as Macroinitiators**

The polymerization of OEGA was carried out using star-like β-CD-*g*-P*t*BA as macroinitiator. β-CD-*g*-P*t*BA (0.01 mmol, 1 equiv.), OEGA (0.6 mmol, 60 equiv.), $CuBr_2$ (0.1 μmol, 0.01 equiv.), $Me_6TREN$ (0.4 μmol, 0.04 equiv.), and DMF (DMF: OEGA=1:1, v/v) were added to a 10 mL Schlenk flask. The solution was bubbled with nitrogen for 1 h to fully remove oxygen, subsequently the sealed reaction vials were placed in a 365 nm UV light reactor for polymerization. At the same time, the external fan was used to cool down the reaction device. After a period of polymerization, the flask was removed to allow air to enter and placed in liquid nitrogen to terminate the reaction. The mixture was then diluted with THF and passed through a neutral alumina column to remove the copper salts. The polymer was precipitated with an excess of cold n-hexane, the precipitate was dried in vacuum at 40 °C for 24 h to obtain star-like β-CD-*g*-P*t*BA-*b*-POEGA.

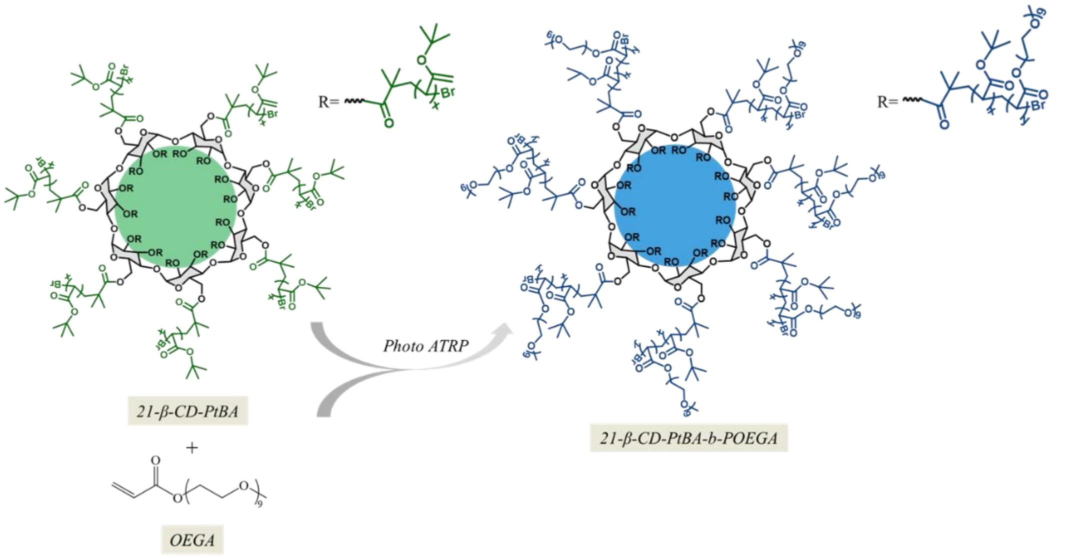


**Scheme S3**. Synthetic route for 21-Br-β-CD-P*t*BA-*b*-POEGA.

**Synthesis of 21-Arm, Star-Like β-CD-*g*-PAA-*b*-POEGA**

The star-like β-CD-*g*-P*t*BA-*b*-POEGA (0.3 g) was dissolved in $CH_2Cl_2$ solvent (30 mL), and then trifluoroacetic acid (10 mL) was slowly added to the above system to hydrolyze the P*t*BA block into polyacrylic acid (PAA) block. The crude product β-CD-*g*-PAA-*b*-POEGA obtained by hydrolysis was concentrated by rotary evaporator and diluted with THF. Subsequently, the polymer solution was precipitated by cold n-hexane three times, the precipitate was collected and freeze-dried for 24 h to obtain β-CD-*g*-PAA-*b*-POEGA.

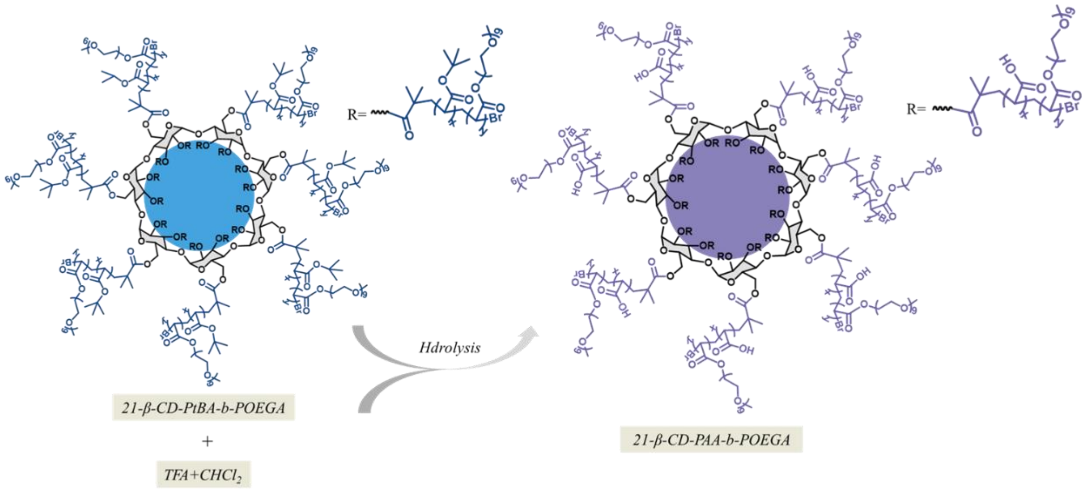


**Scheme S4**. Synthetic route for β-CD-*g*-PAA-*b*-POEGA.

**Synthesis of 21-Arm, Star-Like β-CD-*g*-PAA**

The star-like β-CD-*g*-P*t*BA (0.3 g) was dissolved in $CH_2Cl_2$ solvent (30 mL), and then trifluoroacetic acid (10 mL) was slowly added to the above system to hydrolyze the P*t*BA block into polyacrylic acid (PAA) block. The crude product β-CD-*g*-PAA obtained by hydrolysis was concentrated by rotary evaporator and diluted with THF. Subsequently, the polymer solution was precipitated by cold n-hexane three times, the precipitate was collected and freeze-dried for 24 h to obtain β-CD-*g*-PAA.

**Polymer micelles directed self-assembly of CDs Assemblies**

The CDs (0.5 mg/mL, 0.5 mL) were mixed with β-CD-*g*-PAA-*b*-POEGA (1 mg/mL, 0.5 mL) in deionized water. The solution was mixed and allowed to stand for 24 h to obtain homogeneously assembled solution of CDs assemblies. The obtained solution freeze-dried for 24 h to obtain CDs assembly powder.

**Ion detection**

For the detection of various ions, $FeCl_3$, $FeCl_2$, NaCl, $CuSO_4$, $Gd(NO_3)_3$, $Y(NO_3)_3$, $Tm(NO_3)_3$, $CaCl_2$, $CoNO_2$, $Yb(NO_3)_3$, $MgSO_4$, $Na_2NO$, $PbCl_2$, $CdCl_2$, $NiCl_2$, $BaCl_2$ and $ZnNO_2$ have been used as various ion sources. All chemicals were used as received without further purification. CDs and CDs assemblies solution (0.5 mg/mL, 1 mL) was added into the solutions containing a calculated amount of ions. The PL spectra were recorded after reaction for 10 s. The excitation wavelength was fixed at 365 nm for all the PL spectra.

**Detection of $Fe^{3+}$**

To assess the sensitivity of the fluorescent probe to $Fe^{3+}$, CDs assembly solutions (0.5 mg/mL, 1 mL) were added to solutions containing different iron ion concentrations and PL spectra were recorded after 10 s of reaction. Subsequently, fluorescence spectra were recorded from 380 to 700 nm after each addition at an excitation wavelength of 365 nm. The resulting fluorescence spectra were recorded with a fluorescence spectrometer and the color changes were observed in a closed box under a UV lamp at 365 nm.

**Preparation of CDs Assemblies/Starch Phosphors**

CDs assemblies and starch were mixed at a mass ratio of 1:40 in deionized water. Subsequently, the mixture was sealed in a scintillation vial and stirred continuously for 24 h. The reaction mixture was then centrifuged and the collected precipitate was finally freeze-dried to obtain powder for further experiments.

**Development and Imaging of Latent Fingerprints (LFPs)**

In this study, all fingerprints were collected from the right thumb of male donors. Prior to contacting various substrates, hands were washed with soap and water, followed by gently wiping them on the forehead. Fingers were then pressed onto the substrate, and powder was subsequently sprayed onto the surface, with excess powder brushed away using a small brush. Finally, under 365 nm UV light exposure, yellow fingerprint impressions became clearly visible. The substrates utilized included glass slides, plastic sheets, and stainless steel.

**DLP 3D printing**

A typical procedure for fabricating 3D printed objects is as follows: A 20 mL glass vial was added HEA (5.5 g), followed by $PEGDA_{600}$ (1.66 g), TPO (0.03 g), CDs assemblies (0.2 g), and deionized water (5 mL). Printing parameters concluding Z lift speed and Z retract speed were set to 0.50 mm/s, and the Z lift distance was set to 3.00 mm respectively. The regular cure time per layer (0.2 mm) was 15 s. DLP 3D printer (Anycubic Photon Ultra) with a violet ($\lambda$max = 405 nm) light LED array.

**Fabrication of Yellow-Light-Emitting Film**

PVA (1 g) was dissolved in CDs assemblies aqueous solution (20 mL) following

sonication for 30 min. The transparent CDs assemblies/PVA film was finally got by moulding at 60 °C.

**Characterization**

The molecular weights of the polymers were measured by GPC, equipped with an Agilent 1100, using THF as eluent and PS as a standard sample.$^{1}$H NMR spectroscopy was performed using a Varian VXR-300 spectrometer. $CDCl_3$ and $D_2O$ were used as solvents. FT-IR spectra were recorded by a Magna-550 Fourier transform infrared spectrometer. The morphology of CDs assemblies were imaged by TEM (JEOL 1400 transmission electron microscope (TEM); operated at 120 kV). TEM samples were prepared by placing a drop of assemblies solution on a copper TEM grid coated with carbon (400 mesh) and allowing the $H_2O$ to evaporate in a vacuum oven, and then allowing the sample to dry for TEM imaging. Raman spectra were recorded on Lab Ram HR Raman microscope. The absolute PLQY and the PL decay curves of both solid and liquid samples was measured by FLS1000 using an integrating sphere to ensure the comparability of the test results. Dynamic light scattering (DLS) and Zeta potential measurement was performed on Zetasizer Nano ZS90 (Malvern). Two GPC systems were adopted. One was performed in tetrahydrofuran solution at 35 °C with an elution rate of 1.0 mL min−1 on an Agilent 1260 HPLC system equipped with a G7110B pump and a G7162A refractive index detector. The apparent molecular weights were determined on a single PLgel MIXED-C columns using linear poly(methyl methacrylate) standards. Prior to analysis, the sample was diluted with THF and filtered through a column of neutral alumina to remove the Cu salts, and then filtered through

a 0.22 μm nylon (NY) membrane filter before injecting into GPC columns (inject volume: 20 μL). Another was performed in N, N-dimethylformamide (DMF) solution at 40 °C with an elution rate of 1.0 mL $min^{-1}$ on an Agilent 1260 HPLC system equipped with a G1310B pump, a G1364F refractive index detector and a G1315A diode-array detector. Three TSKgel PW columns in series were calibrated with polystyrene standards. FT-IR spectra were recorded by a Magna-550 Fourier transform infrared spectrometer. Morphology of CDs and assemblies were imaged by TEM (JEOL 1400 transmission electron microscope (TEM); operated at 120 kV). PL spectra were recorded by a LF-1802003 Fluorescence spectrometer. UV-Vis spectra were recorded by as Shimadzu UV-2600 Absorption spectrometer.

## Supplementary Data

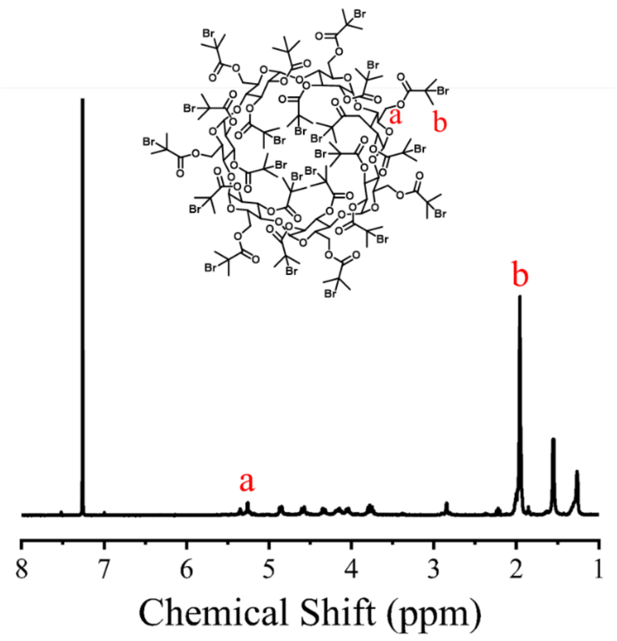


**Figure S1**. $^{1}$H-NMR spectrum of 21-Br-β-CD($CDCl_3$).

The hydroxyl conversion efficiency was calculated using two characteristic peaks of the $^{1}$H-NMR spectrum of the multisite macromolecular initiator 21-Br-β-CD. The peak at the chemical shift δ = 3.5-5.5 ppm (a peak) is indicated as the remaining hydrogen proton peak (49H) on β-CD after the esterification reaction. The peak at chemical shift δ = 1.8-2.2 ppm (b peak), denoted as the methyl proton peak (126H) on the carbon adjacent to Br on 21-Br-β-CD after the esterification reaction. The calculation of the hydroxyl conversion of the esterification reaction can be brought to the equation

$$E_T = \frac{A_b}{18A_a} \times 100\%$$

Where $E_T$ denotes the hydroxyl conversion rate in β-CD involved in the esterification reaction, and Aa and Ab are expressed as the corresponding integrated areas of the hydrogen proton peaks at δ = 5.2-5.3 ppm (a peak) and δ = 1.8-2.2 ppm (b peak),

respectively. After calculation, the value of hydroxyl conversion ET was 99.92%, which indicates that all hydroxyl groups on β-CD underwent esterification reactions to successfully introduce 21 Br atoms as initiation sites for catalytic polymerization reactions.

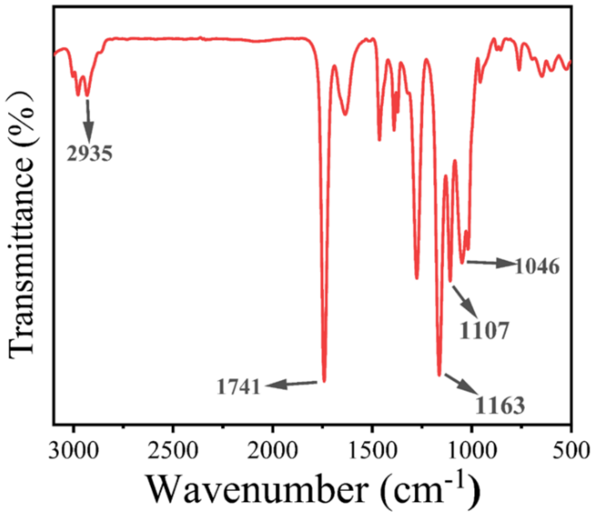


**Figure S2**. FT-IR spectrum of 21-Br-β-CD.

The characteristic peaks of 21-Br-β-CD were mainly found at 2935 $cm^{-1}$, 1741 $cm^{-1}$, and 1163 $cm^{-1}$ from the FT-IR spectra.

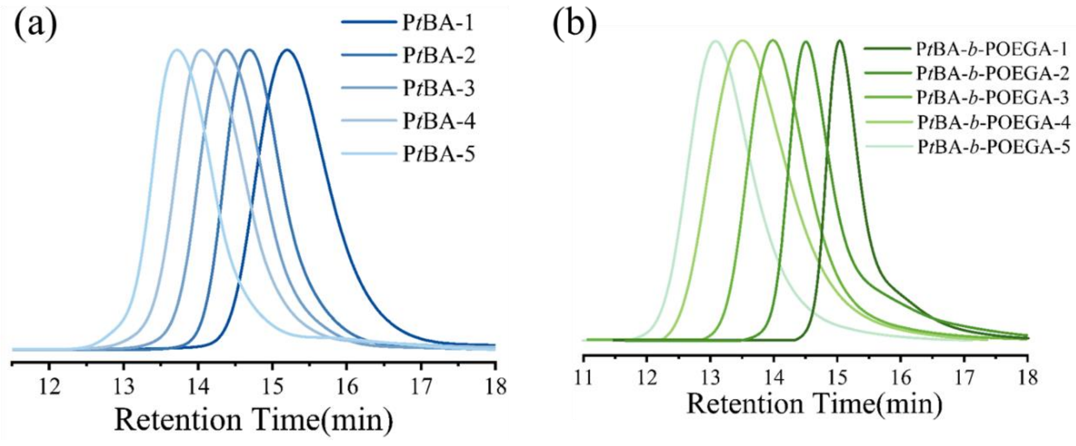


**Figure S3**. GPC trace of (a) β-CD-*g*-P*t*BA and (b) β-CD-*g*-P*t*BA-*b*-POEGA with different molecular weight.

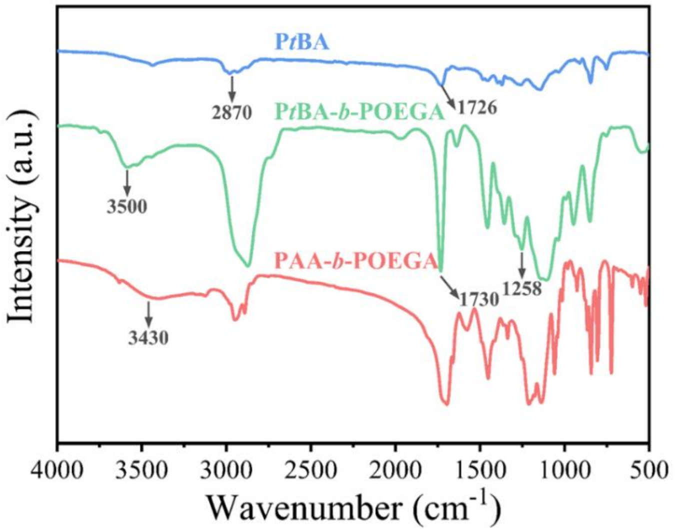


**Figure S4**. FT-IR spectra of β-CD-g-P*t*BA, β-CD-*g*-P*t*BA-*b*-POEGA and β-CD-*g*-PAA-*b*-POEGA.

**Table S1**. GPC and $^{1}$H NMR characterization data of multi-arm star-like β-CD-*g*-P*t*BA.

| Entry | Conv.[a] (%) | $M_{n,GPC}$[b] (kg/mol) | $M_{n,th}$[c] (kg/mol) | $M_W/M_n$[d] |
|---|---|---|---|---|
| 1 | 8.1 | 84.2 | 132.3 | 1.13 |
| 2 | 13.9 | 110.1 | 227.2 | 1.12 |
| 3 | 20.5 | 151.4 | 333.9 | 1.15 |
| 4 | 28.3 | 216.8 | 460.4 | 1.18 |
| 5 | 34.2 | 260.5 | 556.2 | 1.19 |

[a] Calculated from $^{1}$H NMR data.

[b] Number average molecular weight, $M_n$, GPC determined by GPC, calibrated by PS standard.

[c] The theoretical values of $M_n$ calculated from the monomer conversion and the concentration of initiators.

[d] Polydispersity index, determined by GPC.

**Table S2**. GPC and $^{1}$H NMR characterization data of multi-arm star-like β-CD-*g*-P*t*BA-*b*-POEGA.

| Entry | $M_{n,PtBA}$[a] (kg/mol) | $M_{n,POEGA}$[b] (kg/mol) | $M_W/M_n$[c] |
|---|---|---|---|
| 1 | 84.2 | 115.1 | 1.18 |
| 2 | 110.1 | 122.2 | 1.17 |
| 3 | 151.2 | 118.1 | 1.21 |
| 4 | 216.8 | 122.0 | 1.24 |
| 5 | 260.3 | 130.2 | 1.20 |

[a] Date from Table 2, $M_{n,GPC}$(kg/mol).

[b] Number average molecular weight, $M_{n, GPC}$ determined by GPC, calibrated by PS standard.

[c] Polydispersity index, determined by GPC.

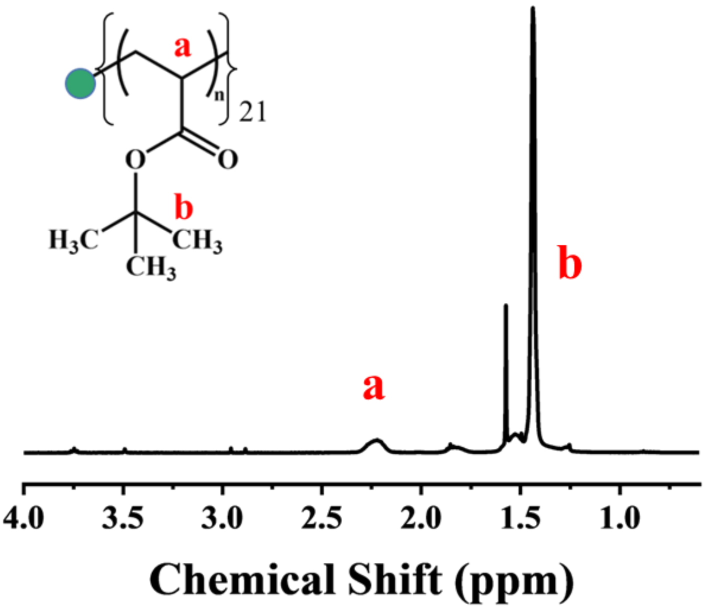


**Figure S5**. $^{1}$H-NMR spectrum of multi-arm star-shaped β-CD-*g*-P*t*BA.

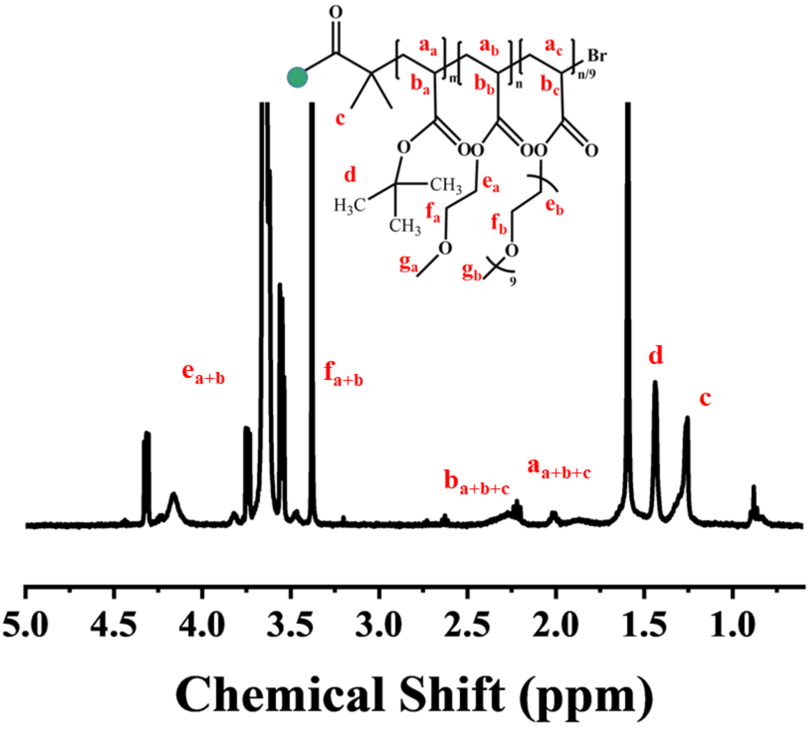


**Figure S6**. $^1$H-NMR spectrum of multi-arm star-shaped β-CD-*g*-P*t*BA-*b*-POEGA.

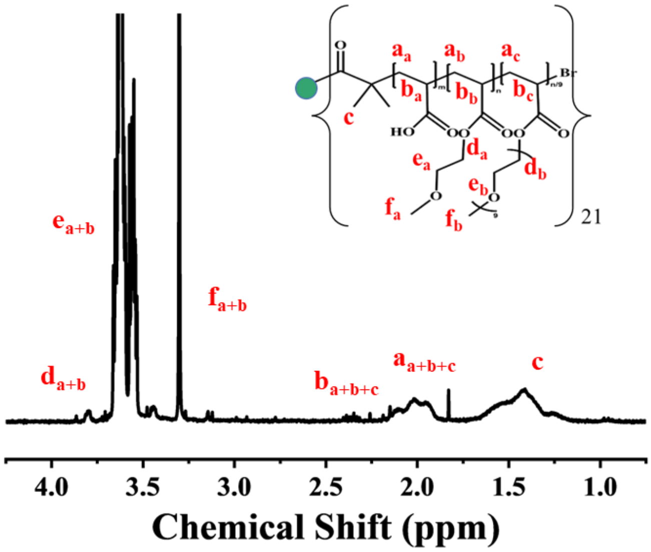


**Figure S7**. $^{1}$H-NMR spectrum of multi-arm star-liked β-CD-*g*-PAA-*b*-POEGA.

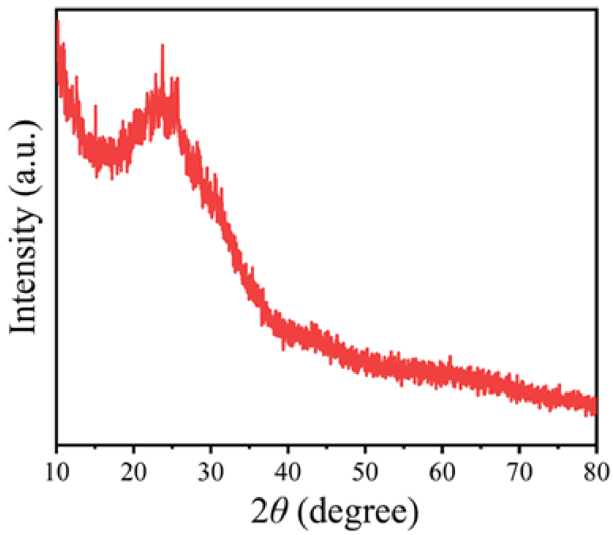


**Figure S8**. XRD pattern of carbon dots

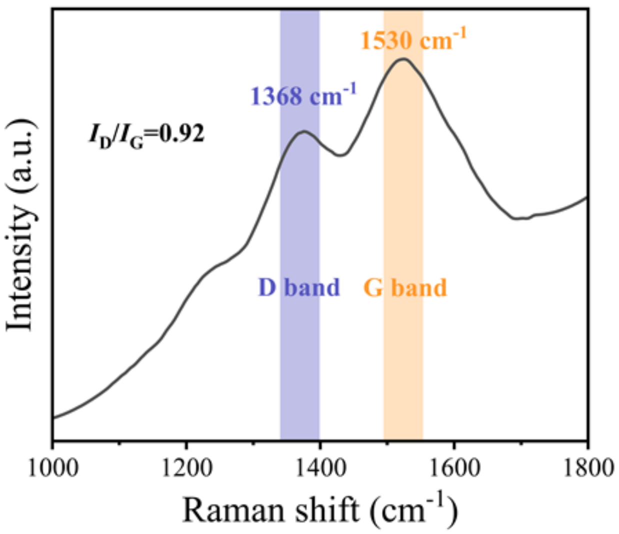


**Figure S9**. Raman spectra of carbon dots.

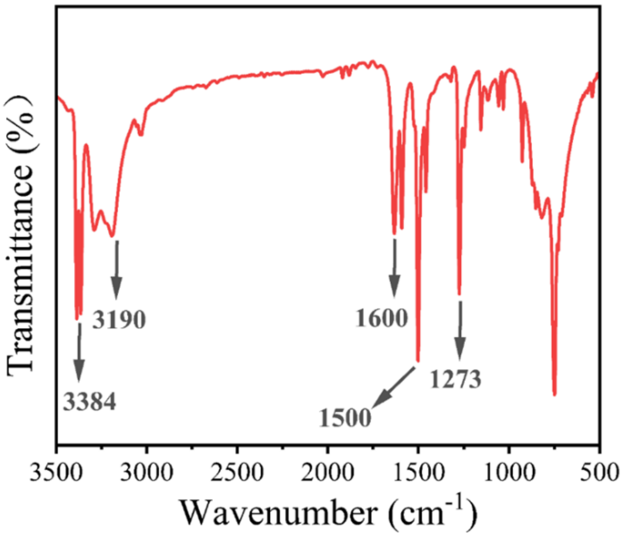


**Figure S10**. FT-IR spectrum of CDs.

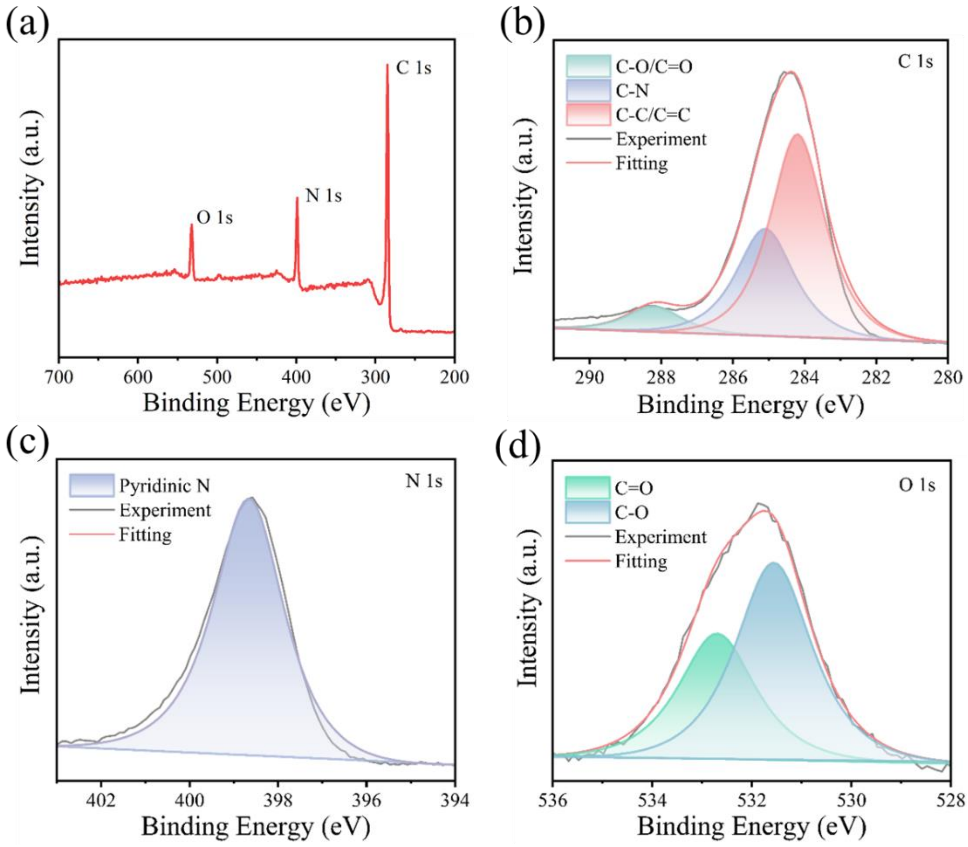


**Figure S11**. (a) Full XPS spectrum of CDs. (b) C 1s, (c) N 1s, (d) O 1s high-resolution XPS fits of CDs.

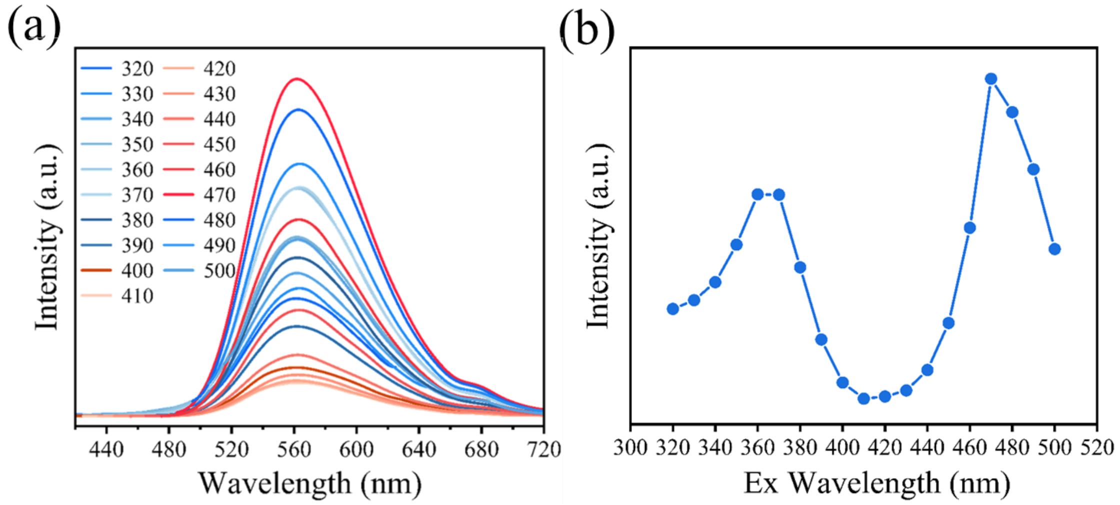


**Figure S12**. (a) Fluorescence spectrum with different excitation wavelengths of CDs solution; (b) Curve of fluorescence intensity at different excitation wavelengths.

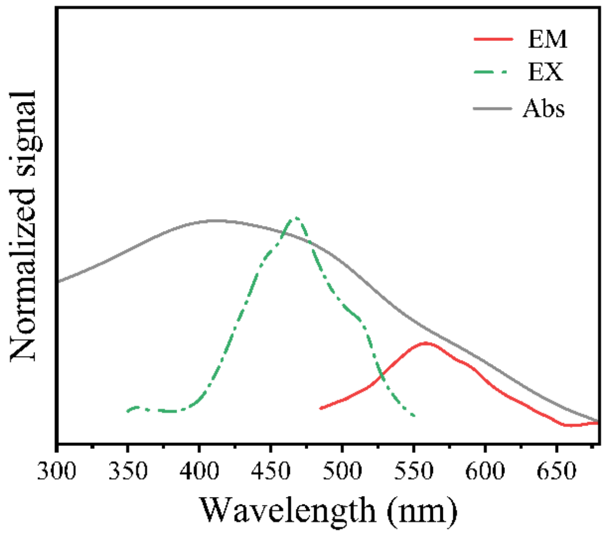


**Figure S13**. UV-vis absorption (gray line), fluorescence emission spectra (solid red lines, $\lambda_{ex}$ = 470 nm), and photoexcitation (green dott lines, $\lambda_{em}$ = 560 nm) spectrum of CDs powders.

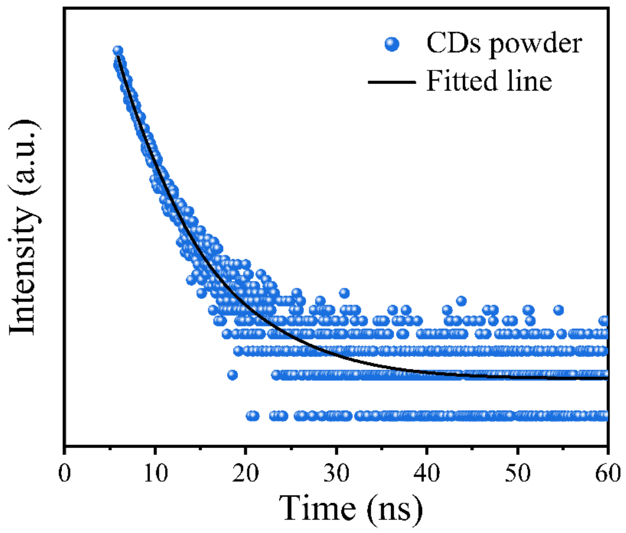


**Figure S14**. Fluorescence decay curve of CDs powder.

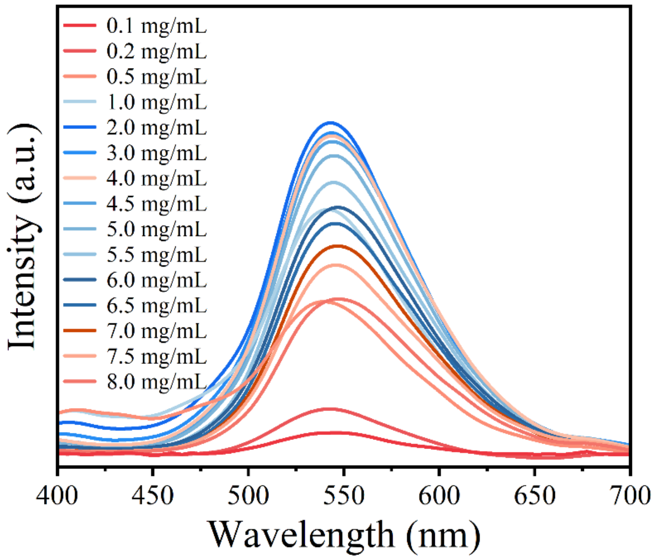


**Figure S15**. Fluorescence spectrum of CDs dissolved in glycerin at various concentrations from 0.1 to 8 mg $mL^{-1}$ ($\lambda_{ex}$ = 365 nm).

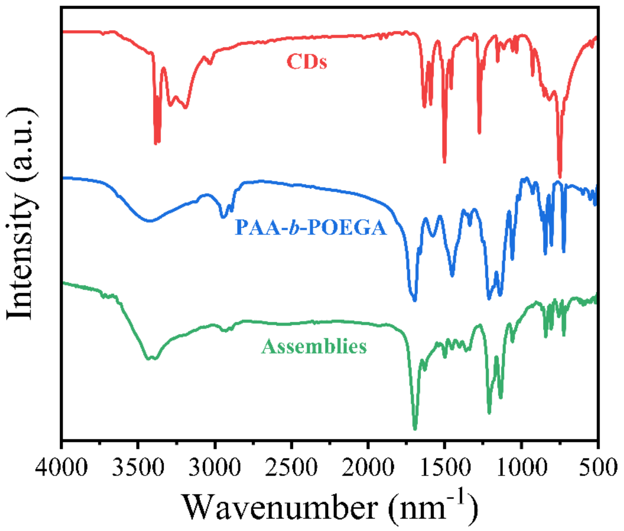


**Figure S16**. FT-IR spectrum of CDs, PAA-*b*-POEGA, and CDs assemblies.

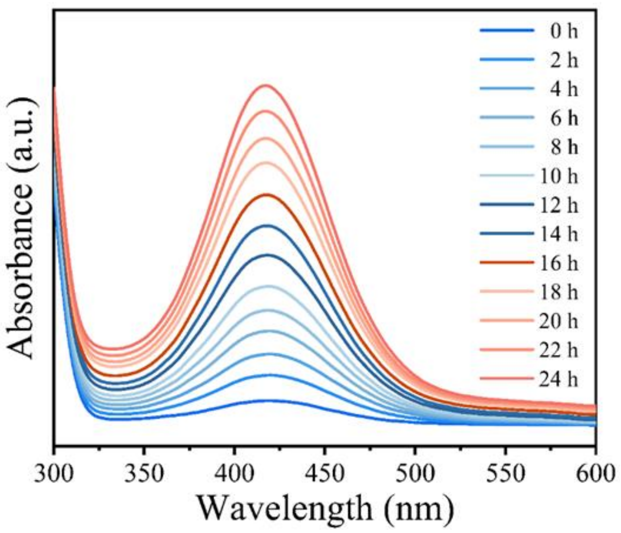


**Figure S17**. UV-vis absorption spectrum of fluorescent CDs assemblies over assembly time.

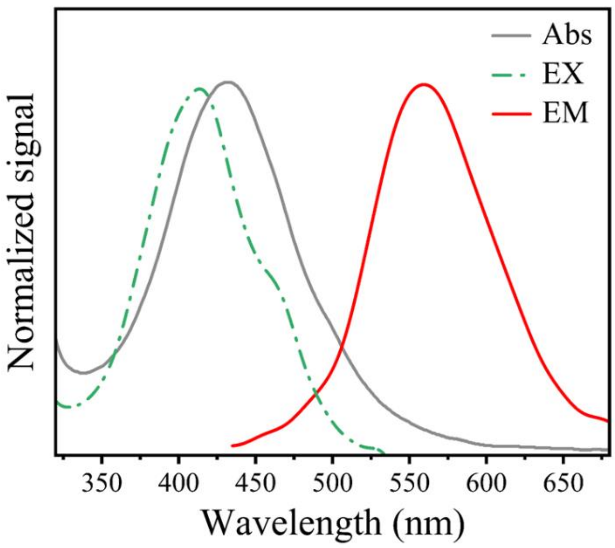


**Figure S18.** UV-vis absorption (gray line), fluorescence emission spectra (solid red lines, $\lambda_{ex}$ = 420 nm), and photoexcitation (green dott lines, $\lambda_{em}$ = 560 nm) spectrum of CDs assemblies aqueous solution.

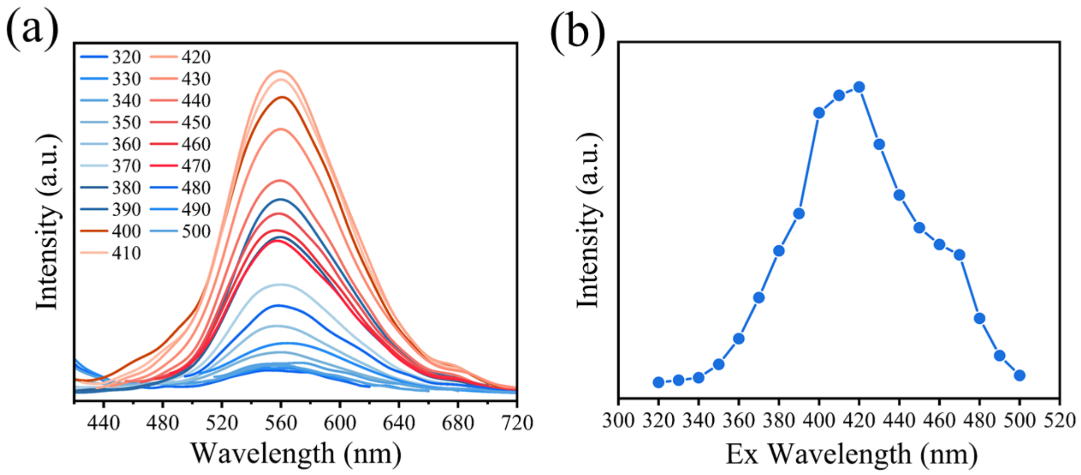


**Figure S19**. (a) Fluorescence spectrum with different excitation wavelengths of CDs assemblies solution; (b) Curve of fluorescence intensity at different excitation wavelengths ($\lambda_{em}$ = 560 nm).

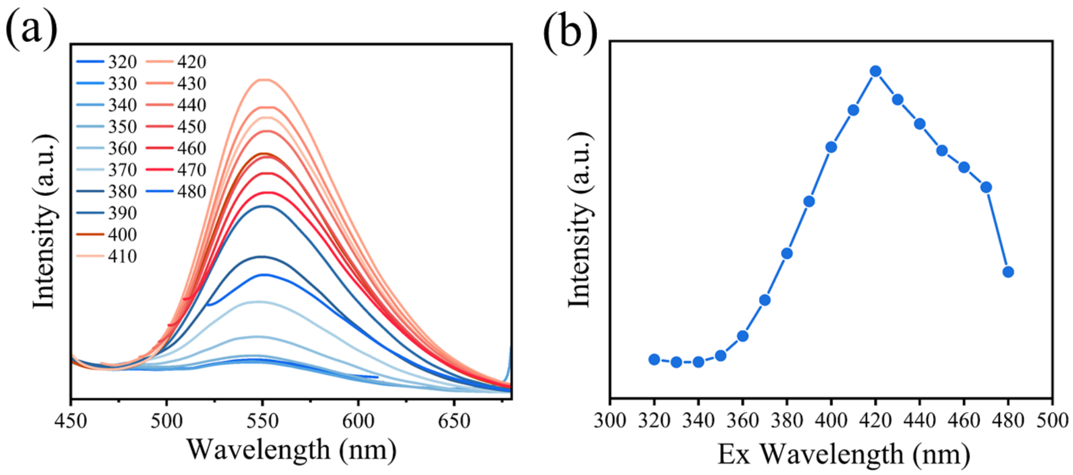


**Figure S20.** (a) Fluorescence spectrum with different excitation wavelengths of CDs assemblies powder; (b) Curve of fluorescence intensity at different excitation wavelengths.

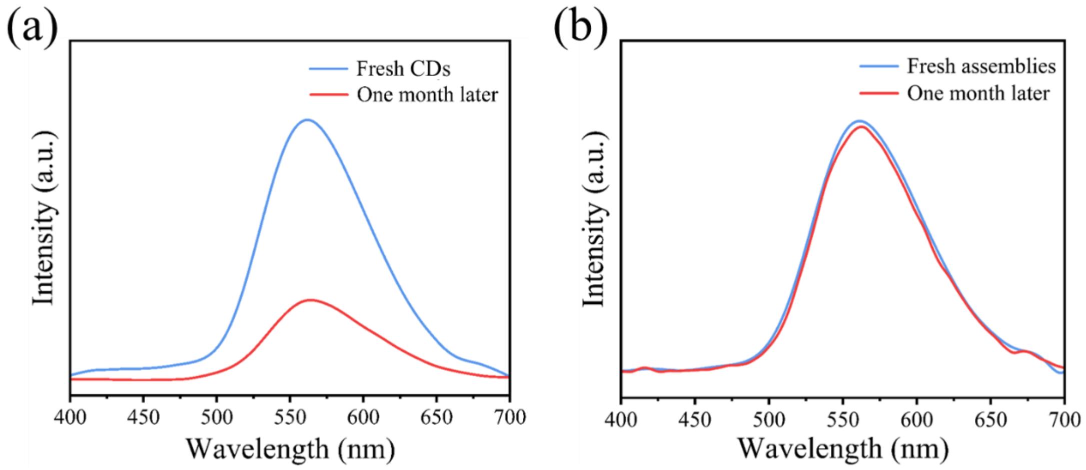


**Figure S21**. Fluorescence emission spectra of (a) CDs and (b) CDs assemblies solution before and after one month of placement.

**Table S3**. A summary of representative solid-state fluorescent CDs has been reported.

| **Materials** | **Methods** | | **$\lambda_{em}$ (nm)** | **PLQY (%)** | **TRPL** | **Ref.** |
|---|---|---|---|---|---|---|
| Trimellitic acid<br>Piperazine | Direct synthesis | Microwave assisted heating | 537 | 58.35 | 5.19 ns | [1] |
| H3BTC<br>KH151 | | Solvothermal method | 410<br>450-530 | 21 | 6.02 ns | [2] |
| Phloroglucinol<br>Urea | | Microwave method | Multi-color | 5.7 – 48.2 | 2.33-6.44 ns | [3] |
| Tween 80<br>Phosphoric acid<br>Sulfuric acid | | RT | 435 | 2.0 | 0.41 ns | [4] |
| Chitosan<br>EDA | | Solvothermal method | 513 | 6 | 5.61 ns | [5] |
| CA<br>DAMO<br>EA | | Solvothermal method | 535 | 9.6 | 5.6 ns | [6] |
| *o*-PD<br>4-dimethylamino pheno<br>$KIO_4$ | | RT | 637 | 16.7 | 5.5 ns | [7] |
| o-PD<br>Acetic acid | | RT<br>Purified via silica column | 308 | 20.2 | - | [8] |
| CA<br>3-fluoroaniline | | Solvothermal method | 676 | 4.17 | 0.38 | [9] |
| MA<br>DTSA<br>Acetic acid | | Solvothermal method | 620 | 5.96 | 4.56 | [10] |
| DSTA<br>HOAc | | Solvothermal method | 580 | 28 | 5.45 | [11] |
| Phloroglucinol dehydrate<br>Boric acid<br>Ethylenediamine | | Microwave method | 565<br>585 | 39<br>31.1 | 3.55<br>2.96 | [12] |
| KHP<br>$NaN_3$<br>BA<br>Formaldehyde | | Microwave method | 432 | 67.8 | 7.15 | [13] |

**Table S3.** A summary of representative solid-state fluorescent CDs has been reported. (continued)

| Materials | Methods | | $\lambda_{em}$ (nm) | PLQY (%) | TRPL | Ref. |
|---|---|---|---|---|---|---|
| *o*-PD<br>Microcrystalline cellulose | Matrix-assisted strategies | Pre-crystallization-controlled | 608 | 24.9 | 2.0 ns | [14] |
| Citric acid<br>Urea<br>$BaSO_4$ | | Microwave<br>Electrostatic assembly | 520 | 27 | 2.02 s | [15] |
| RhB<br>NaOH<br>BA | | Solvothermal method | 513 | - | 30 ns | [16] |
| Phthalic acid<br>Formamide<br>Glycerol | | RT | 525<br>564<br>615 | 20.3 | 4.48 ns | [17] |
| Sublimed sulfur<br>EDA<br>PVA | | Solvothermal method | 423 | - | 9.82 | [18] |
| Tryptophan<br>PVP | | Solvothermal method | 442 | 29.2 | 9.26 | [19] |
| Citric acid<br>Ethylenediamine<br>PVP K90 | | Solvothermal method | 445<br>520 | 83.5 | 14.22<br>15.57 | [20] |
| Citric acid<br>SBA-15 | | Solvothermal method | 410 | - | 7.5 | [21] |
| o-PD<br>4-ABSA<br>Acid | | Solvothermal method | Multi-color | 25 - 72 | 10.91 - 2.49 ns | [22] |
| Citric acid<br>Urea<br>g-$C_3N_4$ | | Microwave method | 520 | 62 | 11.6 ns | [23] |
| Citric acid<br>Urea<br>$AlCl_3 \cdot 6H_2O$ | | Microwave method | 550 | 72.7 | 12.9 | [24] |

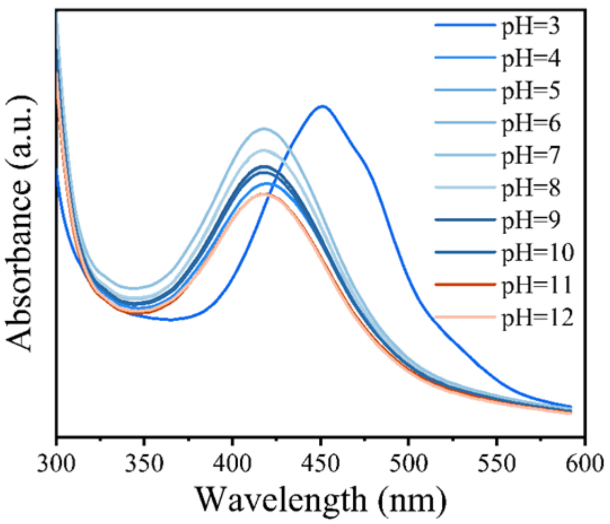


**Figure S22**. UV-vis absorption spectrum of fluorescent CDs assemblies at different pH.

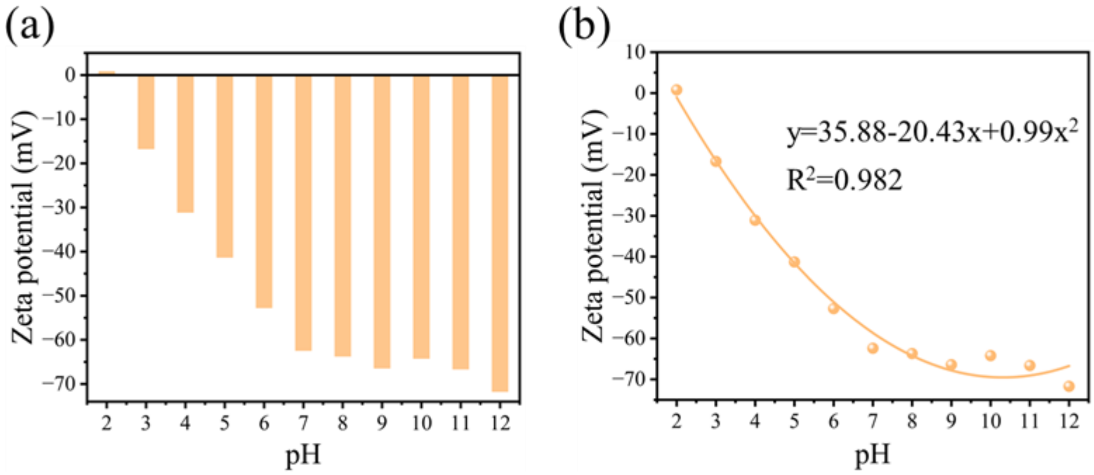


**Figure S23**. (a) Zeta potentials of the CDs assemblies solution with different pH. (b) The relationship between pH and zeta potential.  The concentration of star-liked PAA-*b*-POEGA and CDs are 0.5 mg/mL and 0.25 mg/mL, respectively

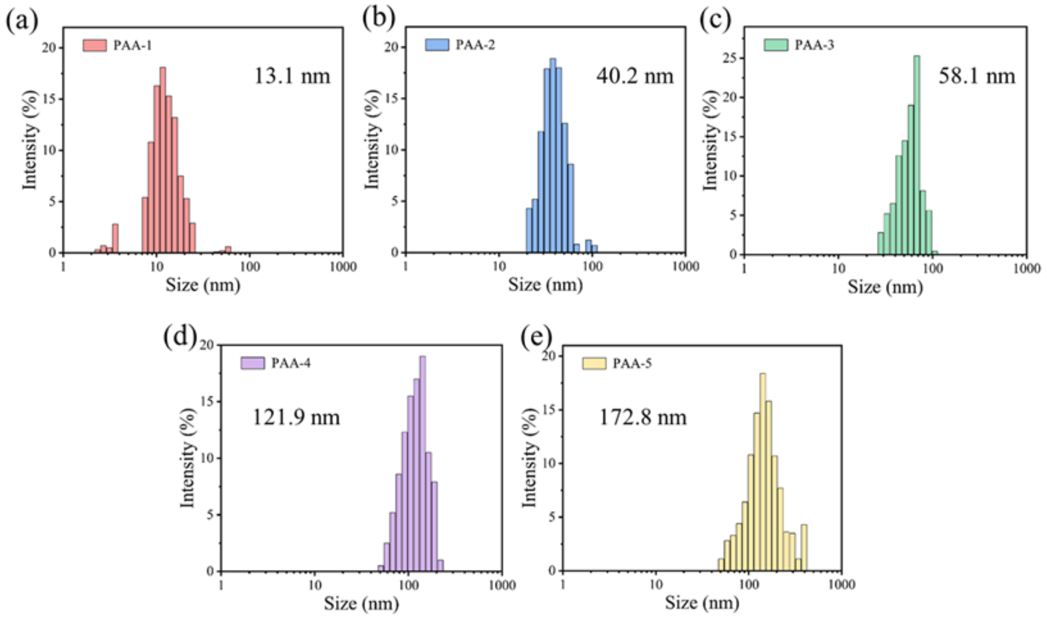


**Figure S24.** (a-e) DLS size distribution of star-liked PAA with different molecular weight. The concentration of star-liked PAA are 0.1 mg/mL.

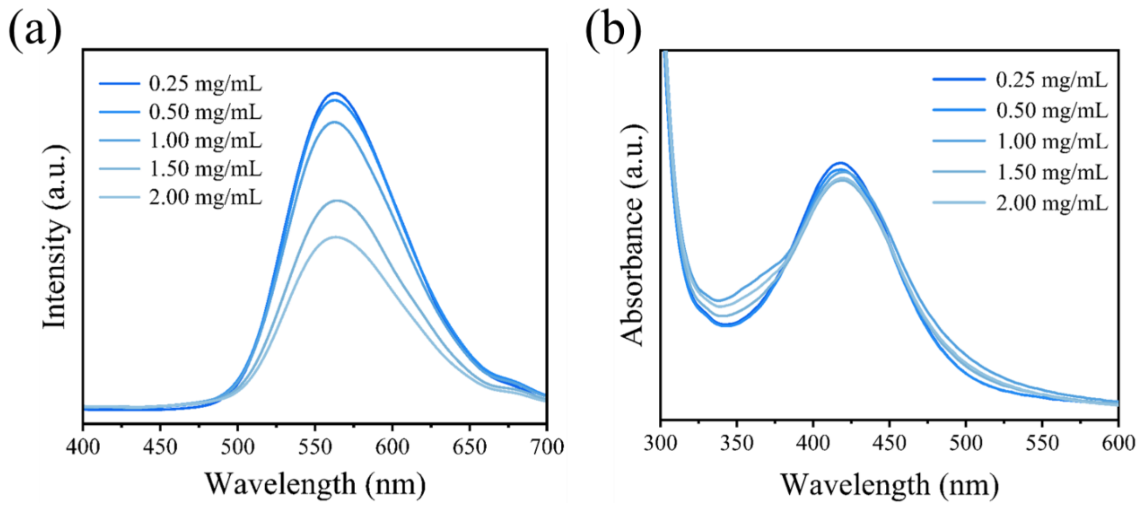


**Figure S25**. (a) Fluorescence spectrum, and (b) UV-vis absorption spectrum of CDs assemblies assembled at 24 h with different concentrations of star-liked polymer templates ($\lambda_{ex}$ = 365 nm).

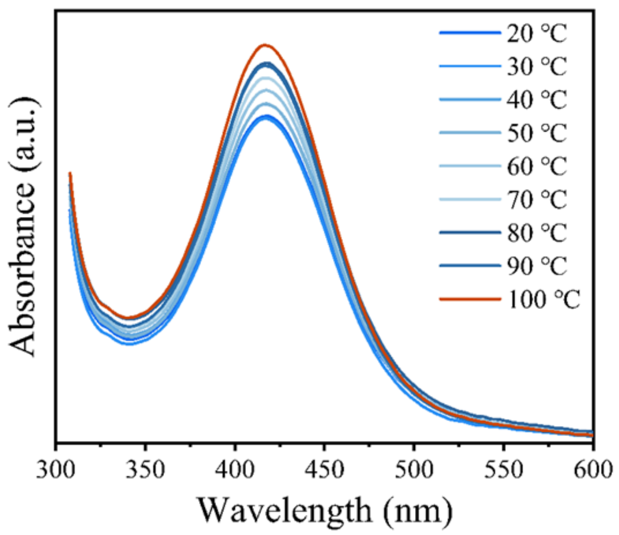


**Figure S26**. UV-vis absorption spectrum of fluorescent CDs assemblies at different temperature.

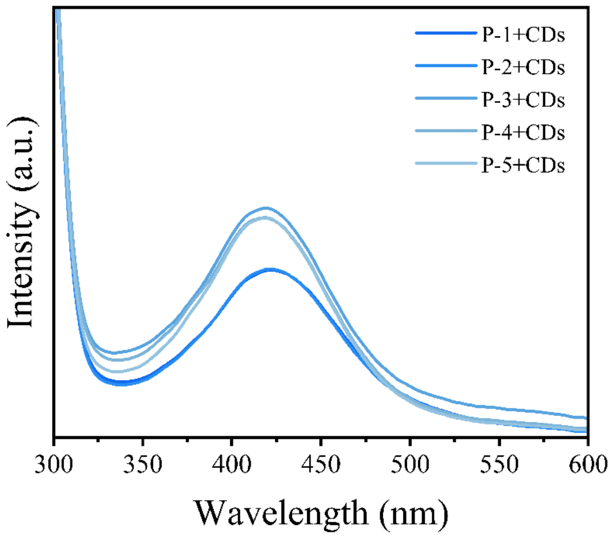


**Figure S27**. UV-Vis absorption spectrum of fluorescent CDs assemblies of different molecular weights.

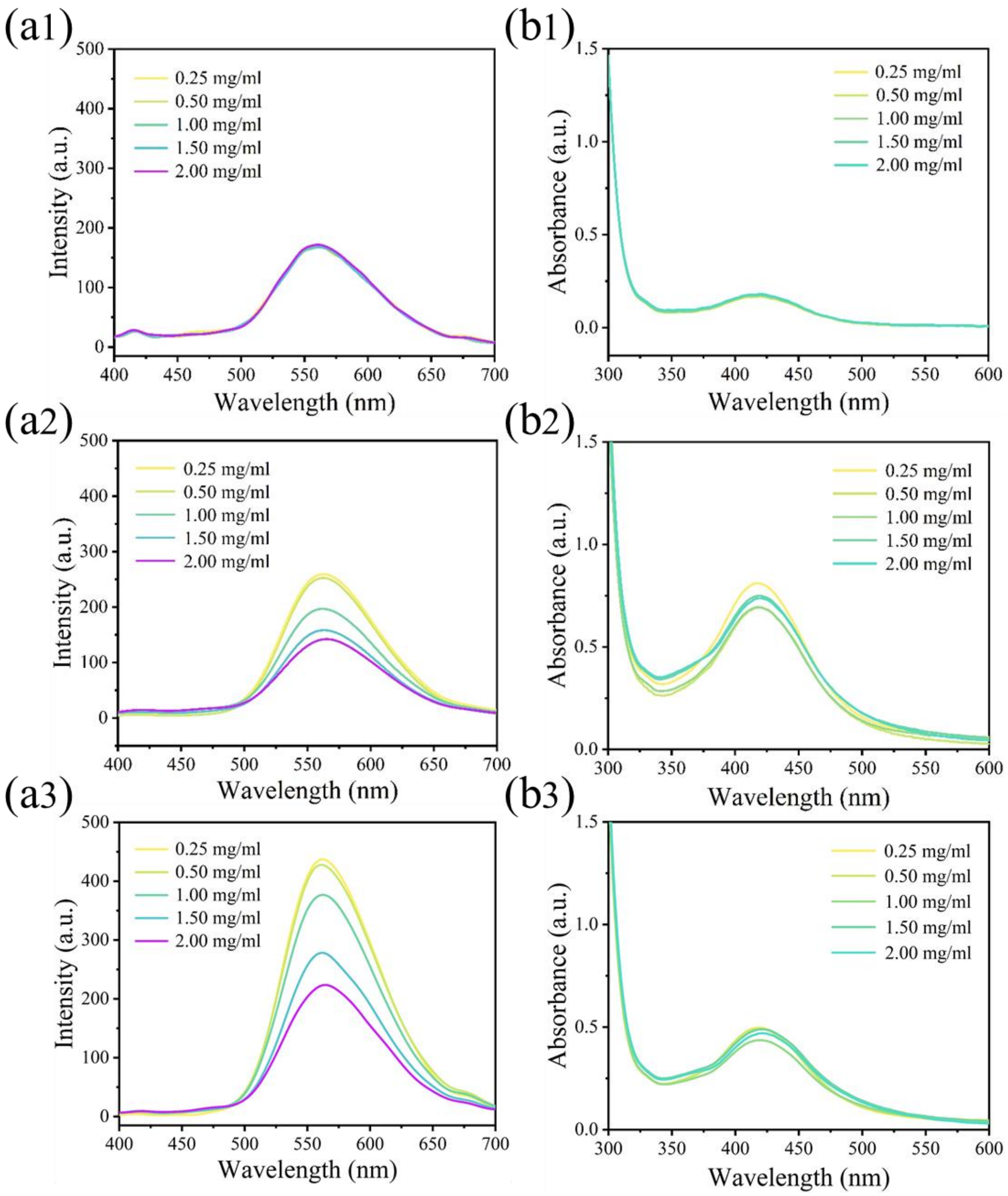


**Figure S28**. (a1-a3) Fluorescence absorption spectrum of CDs assemblies assembled at 0 h, 6 h, 12 h with different concentrations of star-liked polymer templates; (b1-b3) UV-vis absorption spectrum of carbon dot assemblies assembled at 0 h, 6 h, 12 h with different concentrations of star-shaped polymer templates.

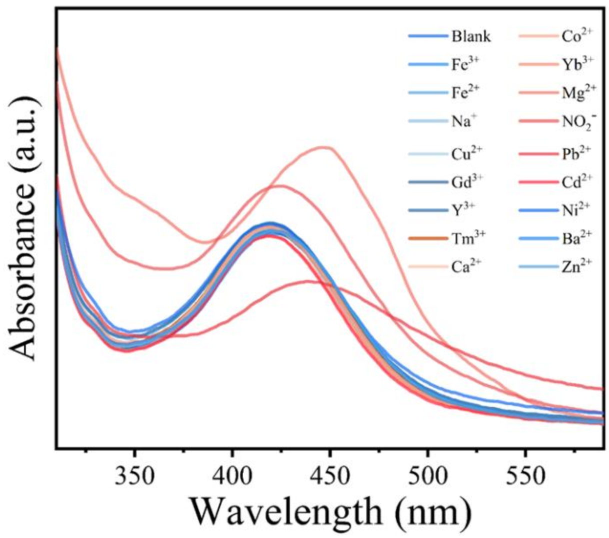


**Figure S29**. UV-vis absorption spectrum of CDs assemblies incorporating different ions.

**Table S4**. Comparison of performance of other fluorescent nano-probes for detection of $Fe^{3+}$

| Detection probe | Ion detected | Detection limit (μM) | Quantum yield (%) | Ref. |
|---|---|---|---|---|
| Y-CDs | $Fe^{3+}$ | 0.127 | 1.17 | This work |
| GQDs | $Fe^{3+}$ | 7.22 | 10 | [25] |
| P-CDs | $Fe^{3+}$, $Fe^{2+}$ | 0.005 | 11.7 | [26] |
| OP-CDs | $Fe^{3+}$ | 0.0073 | 4.29 | [27] |
| CDs | $Fe^{3+}$ | 2.74 | 1.8 | [28] |
| N, P-CDs | $Fe^{3+}$ | 0.84 | 21.7 | [29] |
| N, S-CDs | $Fe^{3+}$ | 0.10 | 24.6 | [30] |

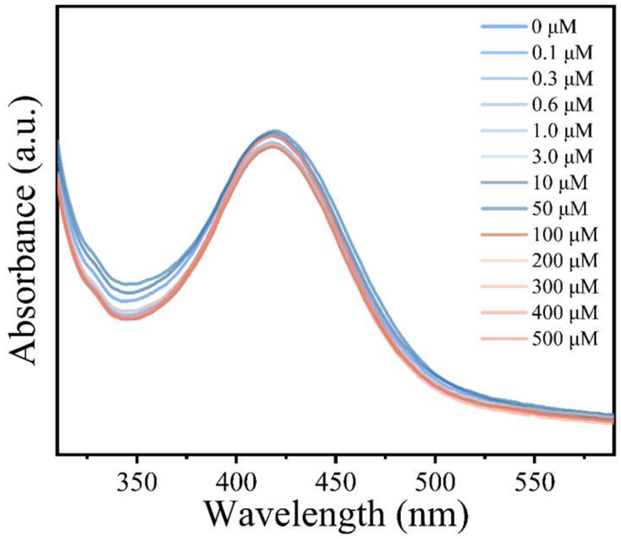


**Figure S30.** UV-vis absorption spectrum of CDs assemblies with different $Fe^{3+}$ concentrations.

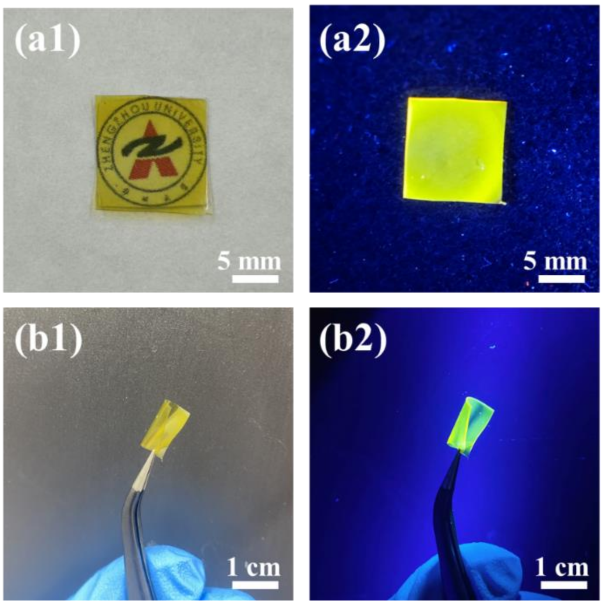


**Figure S31**. Optical image of the yellow-light-emitting CDs assemblies/PVA flexible film under natural light (a1, b1) and UV light (a2, b2).

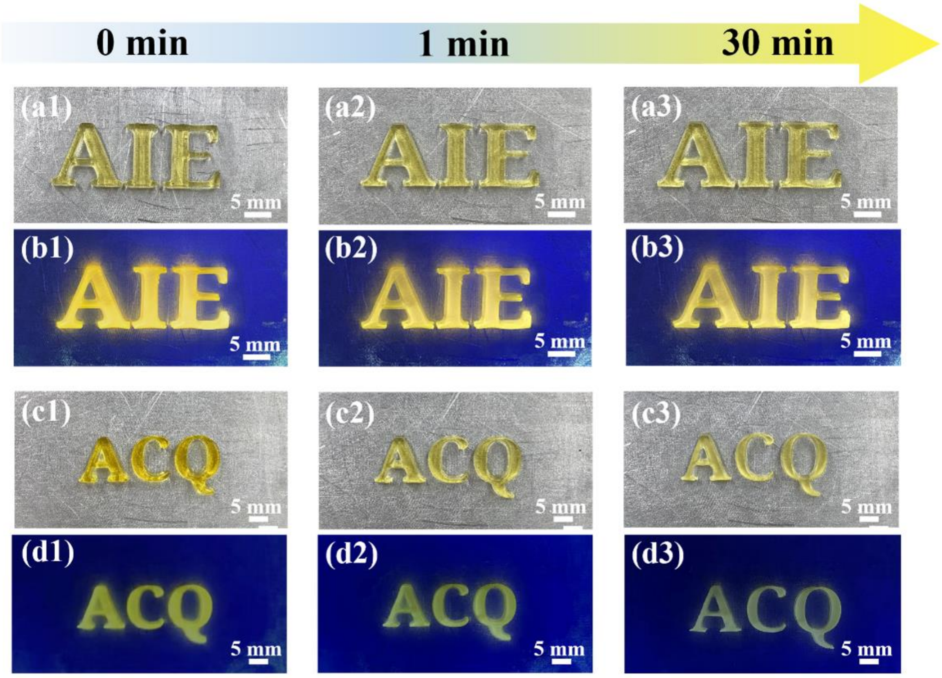


**Figure S32**. Printed "AIE" word with CDs assemblies as luminescent materials under UV irradiation for 0 min, 1 min, and 30 min, respectively, Optical image under natural light (a1, a2, a3) and UV light (b1, b2, b3); Printed "ACQ" word with CDs as luminescent materials under UV irradiation for 0 min, 1 min, and 30 min, respectively, Optical image under natural light (c1, c2, c3) and UV light (d1, d2, d3).

# References

[1] Z. J. Wan, Y. M. Li, Y. Z. Zhou, D. P. Peng, X. J. Zhang, J. L. Zhuang, B. F. Lei, Y. L. Liu, C. F. Hu. High-Efficiency Solid-State Luminescence from Hydrophilic Carbon Dots with Aggregation-Induced Emission Characteristics. *Adv. Funct. Mater*. **2023**, *33*, 2207296.

[2] J. J. Wang, S. F. Zhang, Y. F. Li, C. Y. Wu, W. F. Zhang, H. L. Zhang, Z. Xie, S. Y. Zhou. Ultra-Broadband Random Laser and White-Light Emissive Carbon Dots/Crystal In-Situ Hybrids. *Small* **2022**, *18*, 202203152.

[3] J. L. Wang, J. X. Zheng, Y. Z. Yang, X. G. Liu, J. S. Qiu, Tian, Y. Tunable Full-Color Solid-State Fluorescent Carbon Dots for Light Emitting Diodes. *Carbon* **2022**, *190*, 22-31.

[4] B. P. Jiang, Y. X. Yu, X. L. Guo, Z. Y. Ding, B. Zhou, H. Liang, X. C. Shen. White-Emitting Carbon Dots with Long Alkyl-chain Structure: Effective Inhibition of Aggregation Caused Quenching Effect for Label-Free Imaging of Latent Fingerprint. *Carbon* **2018**, *128*, 12-20.

[5] J. X. Ni, X. L. Huang, Y. B. Bai, B. Zhao, Y. Q. Han, S. Y. Han, T. Xu, C. L. Si, C. L. Zhang. Resistance to Aggregation-Caused Quenching: Chitosan-Based Solid Carbon Dots for White Light-Emitting Diode and 3D printing. *Adv. Compos. Hybrid Ma*. **2022**, *5*, 1865-1875.

[6] L. P. Gao, C. L. Wang, S. H. Xu, P. F. Xia, F. Liu, H. C. Sun, Z. Y. Wang, C. G. Lu, Y. P. Cui. Free Radical-Resistant Carbon Dots for Bulky Luminescent Solar Concentrators with High Optical Efficiency. *ACS Appl. Nano Mater.* **2022**, *5*, 7850-7857.

[7] M. Y. Zheng, H. R. Jia, Zhao, B, C. Y. Zhang, Q. Dang, H. Y. Ma, K. X. Xu, Z. A. Tan. Gram-Scale Room-Temperature Synthesis of Solid-State Fluorescent Carbon Nanodots for Bright Electroluminescent Light Emitting Diodes. *Small* **2023**, *19*, 202206715.

[8] J. H. Xu, Q. J. Liang, Z. J. Li, V. Y. Osipov, Y. J. Lin, B. H. Ge, Q. Xu, J. F. Zhu, H.

Bi. Rational Synthesis of Solid-State Ultraviolet B Emitting Carbon Dots via Acetic Acid-Promoted Fractions of $sp^3$ Bonding Strategy. *Adv. Mater*. **2022**, *34*, 202200011.

[9] H. Z. Ding, J. H. Xu, L. Jiang, C. Dong, Q. Meng, S. U. Rehman, J. F. Wang, Z. S. Ge, V. Y. Osipov, H. Bi. Fluorine-Defects Induced Solid-State Red Emission of Carbon Dots with an Excellent Thermosensitivity. *Chinese Chem Lett*. **2021**, *32*, 3646-3651.

[10] Yang, H.; Liu, Y.; Guo, Z.; Lei, B.; Zhuang, J.; Zhang, X.; Liu, Z.; Hu, C. Hydrophobic Carbon Dots with Blue Dispersed Emission and Red Aggregation-Induced Emission. *Nat Commun* **2019**, *10*, 1789.

[11] F. Liu, S. H. Xu, P. F. Xia, H. Y. Yang, Z. T. Qian, Y. Jiang, Z. Y. Wang, D. Y. Ban, C. L. Wang. Anhydride-Terminated Solid-State Carbon Dots with Bright Orange Emission Induced by Weak Excitonic Electronic Coupling. *ACS Appl. Mater. Interfaces* **2022**, *14*, 5762-5774.

[12] J. L. Wang, Q. Li, J. X. Zheng, Y. Z. Yang, X. G. Liu, B. S. Xu. N, B-Codoping Induces High-Efficiency Solid-State Fluorescence and Dual Emission of Yellow/Orange Carbon Dots. *ACS Sustainable Chem. Eng*. **2021**, *9*, 2224-2236.

[13] H. Y. Li, Z. X. Zhang, J. Ding, Y. Xu, G. R. Chen, J. L. Liu, L. Zhao, N. Huang, Z. Y. He, Y. Li, L. Ding. Diamond-Like Carbon Structure-Doped Carbon Dots: A New Class of Self-Quenching-Resistant Solid-State Fluorescence Materials Toward Light-Emitting Diodes. *Carbon* **2019**, *149*, 342-349.

[14] W. J. Xu, Q. R. Han, C. Y. Ji, F. H. Zeng, X. S. Zhang, J. W. Deng, C. S. Shi, Z. L. Peng. Solid-State, Hectogram-Scale Preparation of Red Carbon Dots as Phosphor for Energy-Transfer-Induced High-Quality White LEDs with CRI of 97. *Small* **2023**, *19*, 202304123.

(15) D. Zhou, Y. C. Zhai, S. N. Qu, D. Li, P. T. Jing, W. Y. Ji, D. Z. Shen, A. L. Rogach. Electrostatic Assembly Guided Synthesis of Highly Luminescent Carbon-Nanodots@$BaSO_4$ Hybrid Phosphors with Improved Stability. *Small* **2017**, *13*, 201602055.

[16] Q. H. Yu, J. H. Jiang, Z. Q. Chen, C. Y. Han, X. M. Zhang, S. Y. Yang, P. Zhou, T. Deng, C. Yu. A Multilevel Fluorometric Biosensor Based on Boric Acid Embedded in

Carbon Dots to Detect Intracellular and Serum Glucose. *Sensors Actuat B-Chem.l* **2022**, *350*, 130898.

[17] Y. N. Zheng, J. X. Zheng, J. L. Wang, Y. Z. Yang, T. P. Lu, X. G. Liu. Facile Preparation of Stable Solid-State Carbon Quantum Dots with Multi-Peak Emission. *Nanomaterials* **2020**, *10*, 303.

[18] G. S. Lv, X. J. Dai, G. M. Lu, L. Ye, G. Wang, L. Zhou. Facile Fabrication of Portable Electrospun Poly(vinyl alcohol)/Sulfur Quantum Dots Film Sensor for Sensitive and Selective Detection of $Fe^{3+}$. *Opt Mater.* **2023**, *135*, 113227.

[19] H. Li, T. T. Xu, Z. Zhang, J. Chen, M. Y. She, Y. L. Ji, B. Y. Zheng, Z. Yang, S. Y. Zhang, J. L. Li. Photostable and Printable Fluorescence Carbon Quantum Dots for Advanced Message Encryption and Specific Reversible Multiple Sensing of $Cu^{2+}$ and Cysteine. *Che. Eng. J.* **2023**, *453*, 139722.

[20] Y. Zhai, X. Bai, H. N. Cui, J. Y. Zhu, W. Liu, T. X. Zhang, B. Dong, G. C. Pan, L. Xu, S. Zhang, H. W. Song. Carbon Dot/Polyvinylpyrrolidone Hybrid Nanofibers with Efficient Solid-State Photoluminescence Constructed Using an Electrospinning Technique. *Nanotechnology* **2018**, *29*, 025706.

[21] Q. Chang, S. S. Yang, C. R. Xue, N. Li, Y. Z. Wang, Y. Li, H. Q. Wang, J. L. Yang, S. L. Hu. Nitrogen-Doped Carbon Dots Encapsulated in the Mesoporous Channels of SBA-15 with Solid-State Fluorescence and Excellent Stability. *Nanoscale* **2019**, *11*, 7247-7255.

[22] L. Wang, W. Li, L. Q. Yin, Y. J. Liu, H. Z. Guo, J. W. Lai, Y. Han, G. Li, M. Li, J. H. Zhang, R. Vajtai, P. M. Ajayan, M. H. Wu. Full-Color Fluorescent Carbon Quantum Dots. *Sci. Adv.* **2020**, *6*, eabb6772.

[23] L. Meng, E. V. Ushakova, Z. J. Zhou, E. S. Liu, D. Li, D. Zhou, Z. N. Tan, S. N. Qu, A. L. Rogach. Microwave-Assisted In Situ Large Scale Synthesis of A Carbon Dots@g-$C_3N_4$ Composite Phosphor for White Light-Emitting Devices. *Mater. Chem. Front.* **2020**, *4,* 517-523.

[24] Y. Y. Zhao, B. C. He, E. S. Liu, J. L. Li, L. M. Wang, S. Chen, Y. Q. Chen, Z. A. Tan, K. W. Ng, S. P. Wang, Z. K. Tang. Aluminum-Based Surface Polymerization on

Carbon Dots with Aggregation-Enhanced Luminescence. *J. Phys. Chem. Lett.* **2021**, *12*, 4530-4536.

[25] A. Ananthanarayanan, X. w. Wang, P. Routh, B. Sana, S. Lim, D. H. Kim, K. H. Lim, J. Li, P. Chen. Facile Synthesis of Graphene Quantum Dots from 3D Graphene and their Application for $Fe^{3+}$ Sensing. *Adv. Funct. Mater.* **2014**, *24*, 3021-3026.

[26] L. Zhou, J. L. Geng, B. Liu. Graphene Quantum Dots from Polycyclic Aromatic Hydrocarbon for Bioimaging and Sensing of $Fe^{3+}$ and Hydrogen Peroxide. *Part. Part. Syst. Char.* **2013**, *30*, 1086-1092.

[27] C. J. Wang, H. X. Shi, M. Yang, Y. J. Yan, E. Z. Liu, Z. Ji, J. Fan. Facile Synthesis of Novel Carbon Quantum Dots from Biomass Waste for Highly Sensitive Detection of Iron Ions. *Mater. Res. Bull.* **2020**, *124*, 110730.

[28] A. Basoglu, U. Ocak, A. Gumrukcuoglu. Synthesis of Microwave-Assisted Fluorescence Carbon Quantum Dots Using Roasted-Chickpeas and its Applications for Sensitive and Selective Detection of $Fe^{3+}$ Ions. *J. Fluoresc.* **2020**, *30*, 515-526.

[29] X. Y. Li, C. Wang, P. Li, X. Y. Sun, Z. Y. Shao, J. Xia, Q. Liu, F. Shen, Y. Fang. Beer-Derived Nitrogen, Phosphorus Co-Doped Carbon Quantum Dots: Highly Selective on-off-on Fluorescent Probes for the Detection of Ascorbic Acid in Fruits. *Food Chem.* **2023**, *409*, 135243.

[30] Q. Du, X. Y. Zhao, X. P. Mei, Y. Q. Zhao, C. Dong, J. F. Li. A Sensitive Sensor Based on Carbon Dots for the Determination of $Fe^{3+}$ and Ascorbic Acid in Foods. *Anal Methods* **2024**, *16*, 939-949.
